# Unravelling the chemical exposome in cohort studies: routes explored and steps to become comprehensive

**DOI:** 10.1186/s12302-020-00444-0

**Published:** 2021-02-11

**Authors:** Sebastian Huhn, Beate I. Escher, Martin Krauss, Stefan Scholz, Jörg Hackermüller, Rolf Altenburger

**Affiliations:** 1 Helmholtz Centre for Environmental Research GmbH – UFZ, Permoserstraße 15, 04318 Leipzig, Germany; 2 Environmental Toxicology, Center for Applied Geosciences, Eberhard Karls University Tübingen, 72076 Tübingen, Germany; 3 Department of Bioanalytical Ecotoxicology, RWTH-Aachen University, Aachen, Germany; 4 Pediatric Epidemiology, Department of Pediatrics, University of Leipzig Medical Center, Leipzig, Germany

**Keywords:** Exposome, Environmental health, Cohort studies, Biomarkers, Mixture toxicity, Biomonitoring, Environmental exposure

## Abstract

Environmental factors contribute to the risk for adverse health outcomes against a background of genetic predisposition. Among these factors, chemical exposures may substantially contribute to disease risk and adverse outcomes. In fact, epidemiological cohort studies have established associations between exposure against individual chemicals and adverse health effects. Yet, in daily life individuals are exposed to complex mixtures in varying compositions. To capture the totality of environmental exposures the concept of the exposome has been developed. Here, we undertake an overview of major exposome projects, which pioneered the field of exposomics and explored the links between chemical exposure and health outcomes using cohort studies. We seek to reflect their achievements with regard to (i) capturing a comprehensive picture of the environmental chemical exposome, (ii) aggregating internal exposures using chemical and bioanalytical means of detection, and (iii) identifying associations that provide novel options for risk assessment and intervention. Various complementary approaches can be distinguished in addressing relevant exposure routes and it emerges that individual exposure histories may not easily be grouped. The number of chemicals for which human exposure can be detected is substantial and highlights the reality of mixture exposures. Yet, to a large extent it depends on targeted chemical analysis with the specific challenges to capture all relevant exposure routes and assess the chemical concentrations occurring in humans. The currently used approaches imply prior knowledge or hypotheses about relevant exposures. Typically, the number of chemicals considered in exposome projects is counted in dozens—in contrast to the several thousands of chemicals for which occurrence have been reported in human serum and urine. Furthermore, health outcomes are often still compared to single chemicals only. Moreover, explicit consideration of mixture effects and the interrelations between different outcomes to support causal relationships and identify risk drivers in complex mixtures remain underdeveloped and call for specifically designed exposome-cohort studies.

## Background

### Rationale and background (benefit and definition of the exposome)

Over the last decades, the prevalence of civilization diseases (e.g. obesity and allergy) increased by a large margin. This cannot be explained by changes in the genetic disposition of single individuals. The rising prevalence of diseases seem rather associated with fast-paced environmental changes [54, 57]. There is an extensive number of environmental factors that may be relevant. To approximate this multitude, the holistic concept of the exposome was proposed by Wild [108] and popularized and refined by Rappaport [81]. Many scientists have followed up on the idea and suggested further specifications for specific sensitive time periods (e.g. pregnancy, perinatal period) [17, 82, 95], selected tissues (e.g. teeth, placenta) [5, 56], focus on specific methodologies [22, 63] or prevalent diseases [83].

The exposome concept aspires to identify chemical exposure, among other environmental factors, relevant for adverse health effects, thus complementing the contribution of life style factors and genomic susceptibility to human disease development [108]. Most exposome definitions acknowledge that typically only chemicals that enter the organism can interfere with cellular or organ functions and provoke adverse effects. Hence, the exposome can be described as the totality of internal human exposure with regards to exogenous chemicals, their biotransformation products, and endogenous chemicals sensitive to various environmental exposures and potentially involved in signaling pathways [30]. This internal chemical environment is highly dynamic and the exposome strives to consider the totality of exposures of an individual over the entire life course from conception until death [81, 85, 108]. It is obvious that the conceptual claim to capture the totality of internal exposure may have practical limitations. For instance, short-lived or reactive chemicals may not be detected, the exposome assessment can be biased by snap-shot samples and/or analytical restriction to available body fluids such as saliva, blood or urine. The endeavor also challenges chemical analytics [78], which need to consider matrix effects for internal exposure, the distribution of chemicals between tissues, and high transformation rates. Moreover, the assessment has to account for sequential exposures and mixture effects [30]. Nevertheless, taking a perspective on the internal exposure seems useful in order to advance from mere associations towards establishing causal links between exposure and effect. Understanding the relation of external to internal exposures, therefore, is a central aspect of an exposome assessment. It allows a revisiting of existing concepts of biomarkers of exposures.

The exposome assessment could represent a critical entity to broaden our understanding of the contribution of environmental factors in the etiology of diseases; it could help to advance the nature-versus-nurture debate. To this end, health data obtained from human cohort studies needs to be evaluated in close linkage to environmental data. Here, especially the integration of various high-resolution cohorts with in depth-phenotyping is aspirational to ensure a sufficient sample size and statistical power. At best, this includes data to approximate a lifetime exposome with all its vulnerable phases beginning as early as the conception, throughout the developmental phases of childhood and adolescence to adulthood and into old age [85]. However, such endeavors are accompanied by challenges such as the sharing and harmonization of data, but also legal and ethical considerations for the use of sensitive human data. Viewed in conjunction with genomic analyses and as part of an overall exposome approach, such data could push us towards the understanding of disease development as well as the prevention thereof. For instance, as many environmental factors are typically subject to regulatory policies, this perspective could ultimately reveal novel points of action for prevention and treatment of civilization diseases [81].

As for now, there is no consensus on how to assess the exposome. From a practical point of view, this is due to the multitude of environmental factors, variation in individual behaviors determining exposures, and the novelty of the exposome concept. Therefore, this review aims to characterize major current projects and their approaches to implement the exposome concept with focus on environmental chemicals.

Major knowledge gaps were identified with regard to the relation between environmental chemical monitoring (external exposure) and human biomonitoring (internal exposure) [24]. In line with this argument, the European Union (EU) funded several projects under the EU Framework Programme 7 (HELIX, EXPOsOMICS, HEALS) in order to advance specific approaches to capture exposome data and link it to health outcomes gained from cohort studies [24]. As the exposome might vary substantially between geographic regions, this review focuses on the analysis of these major European exposome projects with special emphasis on chemical exposure. We, compared the approaches of the European exposome projects to the EU human biomonitoring initiative HBM4EU (https://www.hbm4eu.eu/) and a literature review to summarize current knowledge of associations between human exposures to chemicals and health outcomes. Additionally, this review analyses the chemical and bioanalytical methods used for exposure detection, the aggregation of internal exposures, and novelties related to the association between exposome and health outcome. With regard to the latter, we elaborate on selected aspects of the cohort studies, which were included in the exposome projects.

### Aims and framing of current European exposome studies

As part of this review, projects funded by the European Union under EU Framework Programme FP7 and EU Horizon 2020 were considered in detail. These include the projects HELIX, HEALS, EXPOsOMICS, and HBM4EU [18, 92, 98, 104]. All of these projects related their research to existing infrastructures and data available in different European cohorts with the aim of comparing health outcomes and exposure information [6]. Moreover, they all invested dedicated specific efforts to generate cohort-related biosamples and exposome data.

The Human Early Life Exposure (HELIX) project targeting the characterization of the early-life (pregnancy and childhood) exposome of European populations combined six European birth cohort studies [104]. Research included the characterization and aggregation of external exposures, its integration with internal exposures, and their association with major child health outcomes [104]. Further, it comprised omics data and health outcomes and attempts for simplifying complex exposures into patterns [104].

The Health and Environment-wide Associations based on Large Population Surveys (HEALS) consortium major objective was to advance the methodology and analysis of the human exposome employing advanced statistical tools [92]. The project used data acquired from several current European epidemiological studies including mother/infant pairs, children, and adults [93] to characterize human exposures in conjunction with disease mechanisms and health outcomes [92]. HEALS efforts were built upon human biomonitoring samples, the assessment of exposure biomarkers, and various omics-techniques [92].

The EXPOsOMICS project focus lay on the development of assessment strategies to characterize the mixture exposures to environmental pollutants of consented priority and to approximate the individual-level exposome [98]. To this end, the project utilized data of 12 cohorts including three experimental studies, five mother–child cohorts, four adult cohorts, and subsamples with personal exposure monitoring [98]. Personal and population-level measurements were combined with various omics technologies to characterize biological samples in depth [98].

Finally, the European Human Biomonitoring Initiative (HBM4EU) is funded by the recent EU Horizon 2020 program. The main objective of HBM4EU lies in the coordination and advancement of human biomonitoring efforts across Europe with the ultimate goal to support policy making [14]. HBM4EU is a joint effort of more than 119 institutional partners charged with human biomonitoring tasks and linked third parties, mostly research entities in a total of 30 countries. The project strives at closing knowledge gaps with regard to protocols for detecting and harmonized methods for assessing internal chemical exposures and respective health consequences [14]. Therefore, HMB4EU plans to also investigate health effects in relation to human biomonitoring data with the use of existing cohort studies and biobanks [14].

In summary, the ultimate goal of all projects is to advance the characterization of complex human exposures from environmental and other sources and the association with health outcomes. However, the pursued objectives and chosen approaches are distinctly different between the endeavors. Although exposome and human biomonitoring projects both ultimately aim at controlling environmental risks for human health, they build on different foundations, concepts, and study designs. Exposome projects aim at the totality of environmental exposures and relate dozens to thousands more or less precisely defined signals of exposure to health endpoints in various cohort studies ranging from observational studies to interventions, cross-sectional to longitudinal approaches, and birth up to high age cohorts. Human biomonitoring projects, in contrast, aggregate knowledge to identify comparably few substances of particular concern, derive levels of tolerable exposure to these substances and precisely quantify these using reliable and representative marker compounds.

## Main text

### Exposure analysis strategies

**Fig. 1:**
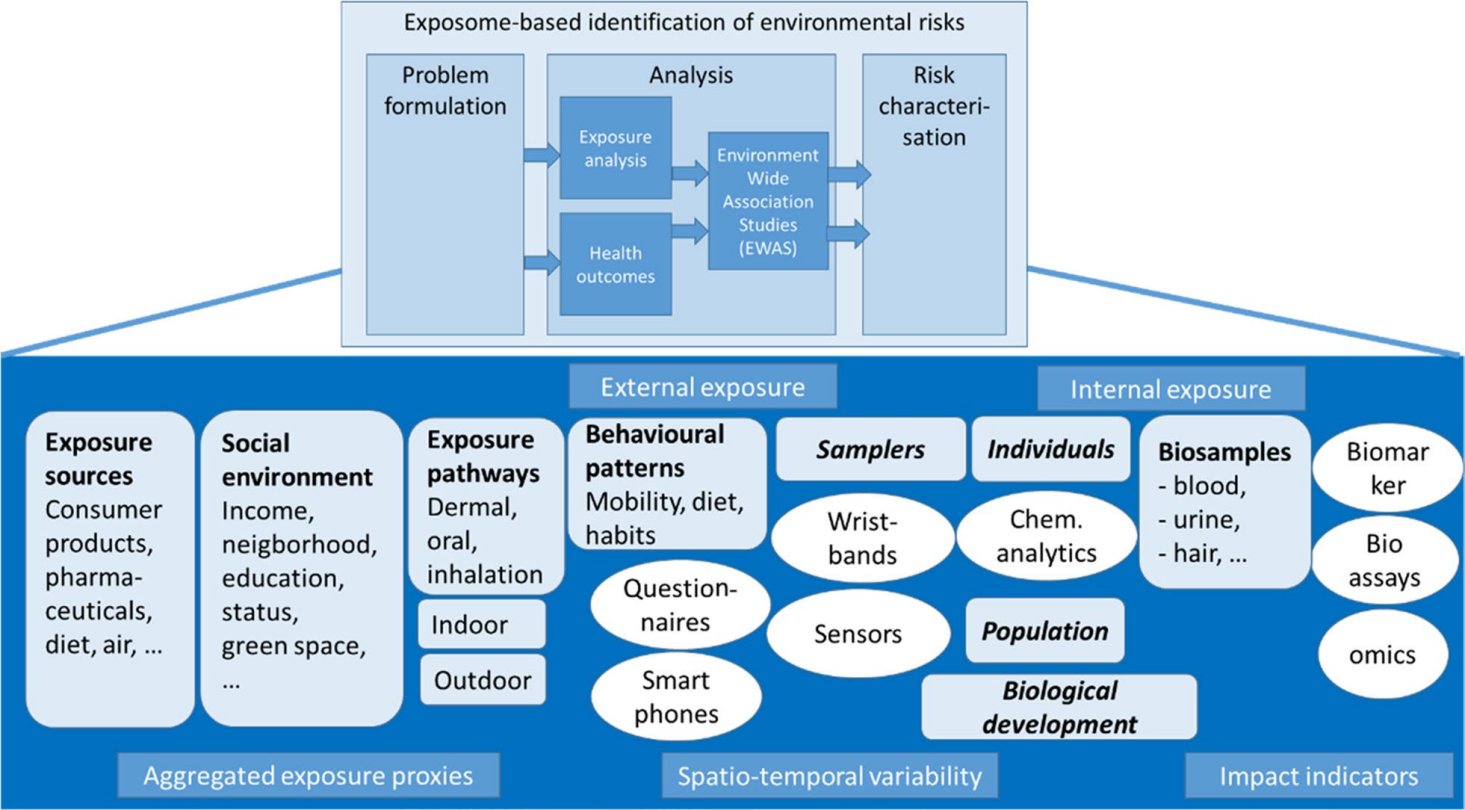
Conceptual framework for exposome studies (top panel), and elements for estimation of multiple environmental exposures against chemicals (bottom panel). Estimation of multiple external human exposures approached from analysis of exposure sources, social environment, exposure pathways, behavioral patterns and immission samplers. Indoor and outdoor environments are often separated in these efforts. Internal body burdens are analyzed in individual or population-based biosamples, such as blood. Typical analytical techniques are indicated in the white egg shapes. All approaches cover different aspects of a comprehensive exposome with regard to aggregation level, spatio-temporal characterization or indication of biological impact

Major decisions on which factors to include in an exposome study, comprise the selection of considered domains of chemical (e.g. pharmaceuticals, food additives, contaminants), physical (e.g. build environment, noise, green space), and social environment (e.g. neighborhood, socio-economic status, infrastructure), as well as biosamples to be used for analysis (e.g. urine, blood, or external proxies). They are central to what we could denominate as the conceptual analysis framework (Fig. 1). Ideally, the framework matches the problem formulation in order to adequately characterize risks. Strategies for the selected exposure analysis may be considered in this context. Technical aspects are decisive in many situations with respect to whether or not an environmental stressor can actually be traced and quantified at the required spatio-temporal scale. Thus, while the exposome concept intends to assess the time- and component-aggregated internal exposure that human bodies experience, many compounds may either not be traceable or are eliminated fast despite elucidating longer-lasting responses. Therefore, exposome research strives to (i) complementary describe external exposure situations more comprehensively to identify potentially unacknowledged stressors, (ii) to relate external to internal exposure to strengthen plausibility of associations between external exposures and observed health outcomes, and (iii) explores options of omics technologies to provide novel, untargeted biomarker detection tools.

The HELIX project, in its goal to provide a global view, developed a multistep approach to characterize various co-exposures in early life including outdoor exposures, individual exposures, and an integration step for external and internal exposure [102]. As their most complex exposure perspective, they defined exposure groups comprising items such as atmospheric pollutants, surrounding natural spaces, traffic, water disinfection by-products, tobacco smoking, lifestyle or socio-economic capital [95] testing whether grouping at this level would already allow suitable simplification of the chemical exposome characterization.

To investigate the notion that the socioeconomic status of individuals could be a major predictor of environmental exposure, a comparative study using data from nine European urban areas attempted to identify reoccurring exposure patterns through co-correlations of the above exposure groups with socioeconomic determinants for urban exposomes during pregnancy [82]. The exposure groups, meteorological factors, air pollutants, traffic indicators, density of the build environment and others were found to correlate at moderate to weak degrees while distance to green space was inversely correlated. Yet, urban exposures of individuals were not found to be associated with socio-economic descriptors (SED), such as education or income of individuals, in this case study of urban populations. Instead social patterning was shown to be of considerable heterogeneity between the different cities [82] and correlation between SED and the exposome was specific for a local area. However, using their exposure biomarkers data from blood or urine for 41 chemical contaminants from six European birth cohorts, it could be demonstrated that specific associations between SED and level of internal contaminant exposure could be discerned [64]. E.g. higher socio-economic position was found associated with certain per- and polyfluoroalkyl substances (PFAS), mercury, arsenic, phenols, and organophosphorus pesticides, while lower socio-economic position was seen associated with cadmium exposure during pregnancy and increased lead and phthalate exposure during childhood [64]. The increased cadmium exposure in pregnant woman could in part be explained by smoking habits.

The HELIX project characterized the exposome for 87 and 122 diverse exposure variables for mothers during pregnancy and their children during development (6–11 years of age), respectively. It was based on six European birth cohort studies, comprising of 1300 mother–child pairs [95]. Among them, about 60 different chemicals were determined in blood or urine samples [43] (see Table 1). On the basis of this data, it was possible to differentiate a pregnancy exposome from the exposome for childhood. At the same time, for some exposure groups such as atmospheric pollutants, surrounding green spaces, meteorology and build environment correlations between pregnancy and childhood exposomes were found, probably reflecting a stable neighborhood for the time domain considered. Furthermore, networks with larger clusters of exposure variables could be described and were interpreted in relation to the predefined exposures groups. Yet, when conducting principal component analysis (PCA) for the pregnancy and the childhood exposome it required 65 of the 87 (75%) and 90 of the 122 (74%) exposure variables to explain 95% of the variance in the original data pairs [95]. Thus, the authors conclude from their collaborative efforts, ‘the early lifetime exposome to be high dimensional in terms of having little redundancy’ [95].

Based on reviewing available approaches for assessing individual exposomes with external measures [58, 97] and internal biomarkers of exposures [92], the HEALS consortium employed a series of analytical, bioanalytical, and computational tools to advance the performance of environment/exposome-wide association studies (EWAS) and tested them with population samples. Biomarkers of exposure are understood in this context according to earlier definitions of e.g. NRC (1987) as indicators signaling exposures from within biological systems or samples.

Regarding the use of modern sensor technologies for chemical exposome assessment, two commentary papers from the consortium suggest that personal location devices, such as smartphone tracking combined with temporal-spatial pollution mapping, promises unique opportunities to gain estimates of an individual’s external exposures against air pollutants such as particulate matter (PM2.5) or nitrogen dioxide (NO_2_) [58, 97]. Furthermore, the project undertook to categorize various types of stressors and reflect the availability of specific biomarkers of exposure and their readiness for use in exposome assessment [92].

The defined stressor categories address different aspects, namely chemical composition (groups of organic and inorganic compounds), physico-chemical properties (persistent and volatile), intentional use such as pharmaceuticals and life style, DNA-damaging agents, context or media of unintentional exposure such as occupational environments, air pollution, food or water contamination. While these categories are certainly not mutually exclusive but overlap, they clearly relate individual stressors to management options. E.g., exposure against volatile organics may be reduced by ventilation, which would not work equally effective for persistent organics; water contamination can be treated in drinking water treatment plants while food contamination must be avoided at the source. Smoking and lifestyle factors can efficiently be dealt with individually. Yet, chemical categories may not be mutually exclusive, e.g. a substance can be volatile and persistent.

Sixty four individual stressors were specified and considered for the different stressor categories, many of them chemical entities. Others address chemical groups such as dioxin-like polychlorobiphenyls (PCB), antibiotics or poorly defined complex mixtures such as diesel exhaust, bio-aerosols or disinfection by-products. The compilation of specific biomarkers of exposure typically comprise chemical compounds of anthropogenic origin or their transformation products detectable in biosample matrices, such as blood, blood serum, blood plasma, urine or breast milk. In total 135 biomarkers of exposure were considered and reviewed with regard to the availability of reference and exposure limit values. Yet, for 12 out of the 64 individual stressors considered, no biomarker of exposure could be retrieved from the literature [92].

Various specific contributions in the field of chemical exposure—human effect investigations, typically based on existing cohort studies or existing biosamples have been provided throughout the HEALS project [44]. The planned integrated use of advanced tools within a European Exposure and Health Survey (EXHES) and the results from the application of the environment-wide association approach to EXHES data have not yet been reported.

The overall ambition of the EXPOsOMICS project was to study the opportunities of novel exposure analysis approaches for strengthening the plausibility between environmental exposures and health outcomes. The project focused on air and water contamination and their health effects during critical periods of life [98]. In particular, the deconstruction of the complex air and water pollution mixtures was pursued as a leading idea.

For personalized detection of exposure of humans by air pollution several routes were followed. The composition of particulate matter with regard to improved size classification, elemental composition and organic and polycyclic aromatic hydrocarbon (PAH) load were differentiated using a novel mobile monitoring design [32]. A portable monitor was coupled with a smartphone app integrating geo-location and other information to provide more accurate individual’s exposure and allow analysis of the influence of microenvironments in contrast to current standard practices of modelled average exposure levels [26]. Personalized air particle samplers apparently still struggle with methodological and sensitivity issues; however, various developments are under way [31] that may render them widely useful in the near future.

To foster the idea that molecules from within the body could indicate past exposure histories that may help in predicting future disease risks [99], the EXPOsOMICS consortium substantially contributed to the Exposome-Explorer [66, 67] developed at the International Agency for Research on Cancer (IARC). The Exposome-Explorer provides an internet-accessible database that summarizes information on biomarkers of exposure related to environmental risk factors for diseases. More than 800 biomarkers have been collated up to date, half of which are directly related to exposure against chemical pollutants, while other reflect e.g. dietary and metabolic components. They are listed in conjunction with metadata characterizing the biomarker structure, methodological information, concentrations in biosamples, and correlations with exposures, use in cohort studies and associations with cancer occurrence.

The development of unbiased exposure biomarkers was sought by employing omics techniques such as adductomics, epigenomics, transcriptomics, proteomics or metabolomics. It is a strategy similar to untargeted chemical analytics to account better for the diversity of pollutants. The omics techniques strive for comprehensive snapshot detection of the respective class of biomolecules and by contrasting different situations they were employed in EXPOsOMICS to obtain fingerprints of specific exposure situations [99]. From the project, success is reported for the task to disentangle contributions from different components of air pollution using metabolome and transcriptome profiles [31]. By contrast, the attempt to discriminate transcriptional and microRNA change patterns for different disinfection by-products (DBPs) exposure was not possible, despite that DBPs may consist of over 700 components. Both types of findings, however, provided additional information employing a combination of top-down (observing complex exposure) and bottom-up (studying component-defined mixture exposures) approaches. This ‘meet in the middle’ approach [99] supported the plausibility between estimated past exposures and observable health effects.

Finally, the perspective of the ongoing HBM4EU project is to provide aggregated indicators of human body burden of chemicals. These summary measures are thought to provide a basis to study and understand variations in time, between countries, sex, age, or socio-economic status and thus provide scope for monitoring of future management activities [18]. The selection process of chemicals, for which monitoring and research activities shall be carried out has been based on expert knowledge around the criteria, relevance of a potential indicator for policy, society and health, as well as consideration of the available biomarker data. The experts were drawn from international, European and national institutions charged with chemical risk assessment and stakeholder consultations. The compound prioritization followed a multi-step procedure, which started with an online survey to nominate substances for further research, included a stakeholder workshop, and several rounds of shortlisting and discussions with stakeholders, national hubs, and the EU Policy Board in order to obtain the final list Thus a transparent strategy was chosen for prioritization of compounds which did not rely solely on scientific evidence, but also societal relevance on a European level. With regard to biomonitoring, the activities subsequently focus on provision and ring-testing of analytical methods for subsequent campaigns within or subsequent to the project. The list of what is called HBM4EU priority substances comprises altogether 18 entries, among them single chemicals or groups of chemicals with related structures, usage pattern or origin. Prioritization, objectives and policy-related questions for each chemical (group) are detailed in two scoping documents in 2016 and 2017–2018 [69, 86].

### Chemicals considered in the EU exposome projects

To provide a structured list of all chemicals considered across the different project, compounds were grouped into broad stressor categories in line with the suggestions made by HEALS [92]. The list of all chemicals, where dedicated analytical efforts were performed within the considered exposome-oriented projects, is collated in Table 1. Persistent organic pollutants (POPs), many of them legacy chemicals and metals, were typically analyzed in human blood samples. Other organic contaminants and current-use pesticides are less persistent and, therefore, often urinary metabolites are quantified in urine samples. Also, volatile organic chemicals cannot be captured in their original form but they may be detected as urinary metabolites.

**Table 1:**
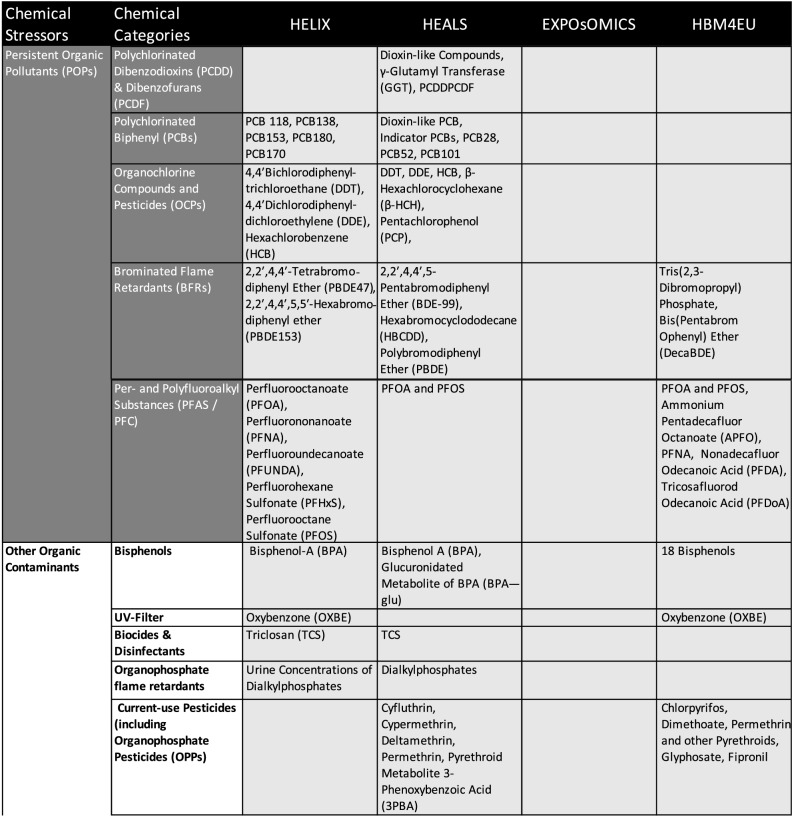

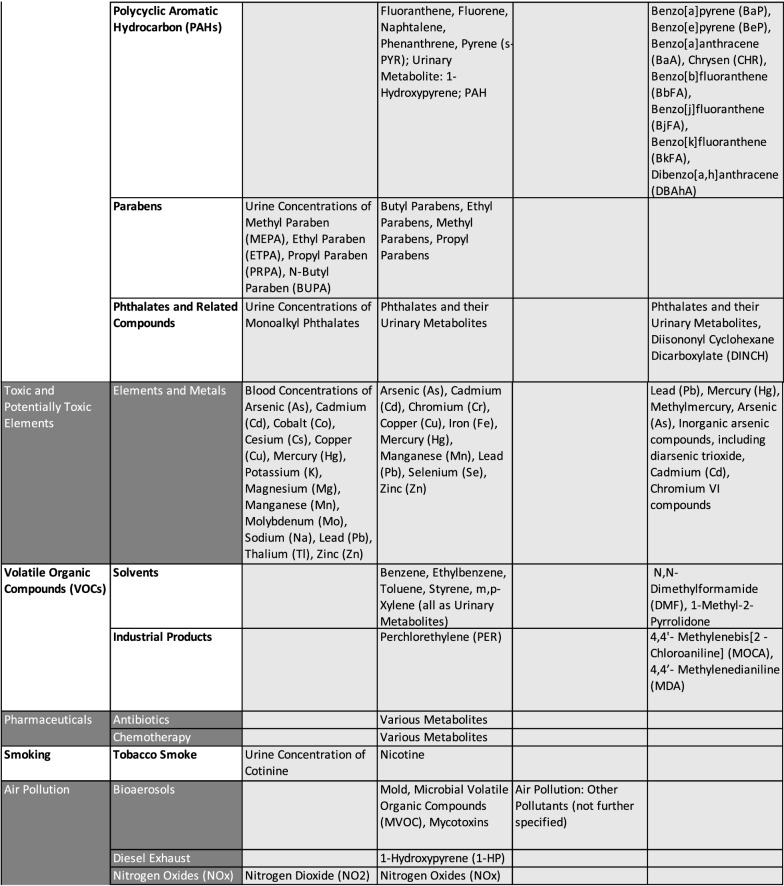

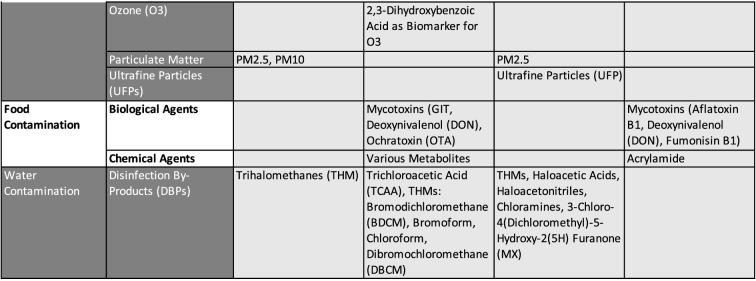
List of chemicals analyzed in the EU projects HELIX, HEALS, and EXPOsOMICS and prioritized chemicals compounds of HBM4EU, HEALS, HELIX

The data in this table is based on following sources: HELIX: [43, 60], HEALS: [92], EXPOsOMICS: [98], HBM4EU: [69, 84]

The four projects described show different priorities regarding the consideration and analytical efforts devoted to different chemical stressor categories. In HELIX, HEALS, and HBM4EU the analyses comprised potentially toxic elements such as lead, mercury, arsenic, and cadmium, various persistent organic pollutants including. e.g. brominated flame retardants, polychlorinated biphenyls, and pesticides, as well as other organic contaminants, such as bisphenols, parabens, and phthalates. HEALS and HELIX furthermore looked at pollution sources such as smoking, air pollution and water contamination. Moreover, HEALS and HBM4EU put emphasis on volatile organic compounds and food contamination. HBM4EU additionally includes research on substances of emerging concern [18, 39, 98]. Finally, HELIX is the only project that explicitly added selected pharmaceuticals.

Thus, the analyses comprised a range of different chemicals including candidates with suspected health effects (e.g. hormonally active substances, carcinogens), chemicals related to air quality and pollution, and, finally, also life style-related exposures (e.g. smoking) nutrition-derived residue and contamination-related exposures.

The chemical selection process can be seen in relation to the project-specific focus. In order to achieve a holistic exposure approach, the HELIX project included the analysis of multiple chemicals instead of ‘one-exposure-one-health-outcome’ perspectives [43]. To meet this ambition, HELIX looked into a total of 59 environmental chemicals comprising 14 essential elements, 15 persistent, and 20 non-persistent environmental chemicals among them pesticides, bisphenols, and phthalates [43].

The HEALS project put focus on substances with known particular relevance to human health in Europe [92]. They based the selection of chemicals on an expert-driven process, which included, among others, national and EU level policy stakeholders but also scientific partners. This resulted in a list of 98 analytes, which can be attributed to nine of the stressor categories ranging from elements via organic pollutants to food and water contamination.

The EXPOsOMICS project focused on contaminations of air and water with the aim to provide data on a broad spectrum of chemicals and chemical mixtures [98]. In particular, for air pollution, EXPOsOMICS analyzed particulate matter and ultrafine particles, while for water contamination they focused on disinfection by-products [98]. Here, mixtures were acknowledged with the aim to identify risk drivers.

In HBM4EU, several international and European human health risk assessment schemes were evaluated (e.g. WHO, FAO) with the aim to implement a prioritization strategy [14, 84]. The first group of priority substances was described in 2016 [84] and was complemented by a second list in 2018 [69, 84]. The priority groups were clustered according to their toxic potency, for modes of actions regarding different cellular systems, common target organs, or common phenomenological effects [29]. Also, adverse outcome pathways (AOP) like fatty acid composition changes in liver, decreased anogenital distance and cranio-facial malformation were used for grouping [14]. The concept of AOPs aims at linking external exposures to respective cellular concentrations and biochemical mechanisms that ultimately lead to responses at the level of organs and organisms but finally also at population levels [30].

The array of substances, which have been collated from the described exposome projects, will subsequently be compared against other biomonitoring efforts as well as against literature-based knowledge about chemicals that have been reported in connection with elucidation of health effects.

### Association of exposome and health outcomes

One of the biggest challenges of the exposome approach lies in establishing robust relationships between multidimensional environmental data and health outcomes acknowledging the “extremely complex multistage development process” [102].

To cover health-relevant data throughout the entire life course, it is crucial to include cohort studies that cover the entire age range, that are representative for the general population (including all sexes, socio-economic strata, and ethnic groups), and follow, at best, a prospective, longitudinal design [85, 109]. The cohorts included in the European exposome projects cover several of these ambitions, yet are still too few to allow generalizations (for a detailed overview of study characteristics, please see Additional file 1: Table S1). The study designs included cross-sectional surveys, longitudinal cohort studies, and interventional trials with an age range from 0 to > 80 years with sample sizes from 30 to 14,000 participants [50, 112]. While HELIX focused on childhood with the inclusion of birth-cohorts [104], HEALS, EXPOsOMICS, and HBM4EU cover the entire life span with a stronger focus on adulthood [93, 94, 98]. The gender ratio across the majority of studies is balanced with exceptions that focus on females [62, 94]. Another relevant aspect is the respective time of enrollment, which starts as early as the 1990s (e.g. ALSPAC, [36]) and is still ongoing in other studies (e.g. ENVIRonAGE, [49]). This is of major relevance as the overall exposure of a child born in the 1990s will clearly differ from a child born nowadays. To name only a few buzzwords indicating those changes: smartphones, non-smoking protection laws, gas exhaust standards, a changing climate, and the coronavirus pandemic.

An expansion of the endeavor to link the (chemical) exposome to health outcomes is highly desirable. However, the inclusion of more cohorts into one overall project poses several challenges. These include not only methodological issues, such as the harmonization, inventory, and cataloguing of data, but also comprise legal and ethical challenges with respect to personal data and respective data protection laws. Furthermore, it poses a series of statistical issues. Amongst others they have to cope with the large overall number of exposure variables, missing data, the correlational structure within exposure and health data, interaction- or mixture effects, and biases in multi-center studies. Not surprisingly, therefore, all exposome projects considered in this review strived to systematically summarize, evaluate, and advance statistical methods for building exposome-health associations [7, 85, 87, 97]. While significant progress has been made for variable selection and identification of pairwise interactions between exposures, the untangling of relevant and confounding exposures and the analysis of exposome data in longitudinal studies remain challenging [21, 55, 85].

Throughout the described exposome projects, a large amount of data has been collected. Exposures related to lifestyle factors include e.g. maternal stress [73], tobacco smoking [40, 72], or occupational exposure to chemicals [11] but also air pollution. Regarding chemicals, HEALS reported on the analysis of 98 chemicals [92], HELIX included 59 [43, 60], and EXPOsOMICS seven substances [98]. In HBM4EU 230 compounds are considered as potentially relevant for human biomonitoring [18, 84]. However, stating the exact number of analyzed chemicals is ambiguous as the projects sometimes do refer not only to single entities (e.g. lead, selenium, mercury volatile organic compounds, and various organic pollutants, see Table 1) but rather to substance groups (e.g. benzophenones, trihalomethanes), which potentially contain multiple compounds.

On top of these already diverse data, many cohort studies additionally complement their analyses with biomarkers gained from biospecimen (e.g. blood, urine) such as enzyme levels and activities, hematological markers [52], hormone levels, metabolite levels (metabolome) or DNA adducts (adductome, [80]) such as DNA methylation levels [107]. Metabolome and transcriptome analyses, e.g., were performed in HELIX, HEALS, and EXPOsOMICS, while epigenomics and proteomics were done in HELIX and EXPOsOMICS only [92, 98, 104] and the projects HEALS and EXPOsOMICS, furthermore, completed adductomics [92, 98]. As they are interpreted in different directions it is not straightforward to summarize their added value. Biomarkers may be seen to aggregate exposures of different substances that hit the same target. This would support a comprehensive exposome detection. They can, however, also be employed to support causality between exposure and outcome by looking at them as effect indicators for downstream biological responses, or they are used to explain observable variation within populations through different susceptibilities of subgroups.

In practice, the exposome projects, which were considered in this review, pursued different approaches to address external and internal exposures in their studies and relate these to a variety of health outcomes. For an overview of the addressed health outcomes see Table 2 and for characteristics of the used cohort studies refer to Additional file 1: Table S1.

**Table 2:** Health outcomes assessed in the EU exposome projects

Health outcome	HELIX	HEALS	EXPOsOMICS	HBM4EU
Allergies/asthma	Yes	Yes		
Neurodevelopment	Yes	Yes		Ongoing
Weight development	Yes	Yes		
Obesity	Yes	Yes		
Type 2 diabetes	Yes			
Neurodegenerative diseases	Yes			
Cancer	Yes		Yes	Yes
Acute coronary events and blood pressure			Yes	

Please refer to Additional file 1: Table S1 for a detailed overview regarding the characteristics of the used cohort studies including study design, sample size, age, time of enrolment, and gender ratio

HELIX undertook a variety of outcome assessments starting with one-exposure-one-outcome assessments to elaborate benefit-harm scenarios in order to cover different complexities of exposure scenarios [104]. These results were documented in various publications on blood pressure during pregnancy and in children [105, 106], birth weight [68], fetal growth [2], lung function in children [3], and childhood obesity [103]. For this, HELIX used data of six European birth cohort studies with a total of 32,000 mother–child pairs with initial recruitments ranging from 1999 to 2007 and a representative gender ratio [104]. Additionally, two subsamples to assess biomarkers in mother–child pairs (*N* = 1.200) and a nested repeat-sampling panel study (*N* = 150) have been established [104]. Overall, HELIX contributed to demonstrate the feasibility of a harmonized, large-scale exposome wide association study and advanced standardized exposure assessment especially in the urban context [46]. This includes, among others, methodological developments, such as more efficient statistical approaches, the generation of a complete molecular profile data set, and improved measurement of a variety of exposures as e.g. air pollution, built environment, and green spaces [46]. More specifically, HELIX increased the overall reliability of findings as they based their analyses on the assessment and statistical testing of a large number of exposures. This allowed for discerning confounding co-exposures and account for multiple testing [46]. HELIX identified air pollution (in relation to infant mortality) and secondhand smoke (in relation to asthma) as the largest negative contributions with regard to child health. Furthermore, they confirmed potential hazards from factors such as dampness, formaldehyde, and ozone and their associations with childhood asthma and respiratory symptoms, as well as lead in association with mild mental retardation [46]. Moreover, with the comprehensive generation of molecular profile data sets (including urinary and serum metabolomics, plasma proteomics, blood cell DNA methylation, transcriptomics, and microRNA data) for a subgroup of 874 children, HELIX was able to conduct an Exposure Wide Association Study (ExWAS), which identified several clusters by association. These were used to identify exposure sources, e.g. fish/seafood as source of polyunsaturated fatty acids, and also metal contaminations [46].

HEALS, so far, published work on multiple health outcomes including (gestational) weight gain [62, 100], birth weight [11], psychomotor development [70, 71, 74], fine motor skills [76], neurodevelopment [72, 73, 89], and allergy and asthma [40]. To that end, HEALS used data of at least 17 different cohort studies, which involve birth cohort studies, national registries, screening trials, and adults with sample sizes from 186 to > 130,000 and an enrolment as early as 1992 and up to 2011. The information on gender that the authors were able to verify was available for only a few cohorts from the publications. Major achievements of HEALS include the establishment of large, harmonized exposure and health databases, the advancement of EWAS methodologies including the linkage with omics data, data mining techniques, and machine learning [45]. Further milestones comprise reliability testing and validation of personal and remote sensors for individual exposure in five European countries [45]. In detail, HEALS applied a life-course approach that is based on existing data and characterized the external exposures of about 550 individuals. This demonstrated as proof of principle the applicability of the exposome methodology to existing studies [45]. As a first step in establishing individual ‘life-long multi-stressor exposure profiles’, this might improve preventive strategies or assist policy-makers [45].

Up to now, EXPOsOMICS yielded several publications relating outcomes such as arterial blood pressure [37], cardio- and cerebrovascular diseases and events [34, 91], natural-cause mortality [9], cardiovascular mortality [10], nonmalignant respiratory mortality [25], and lung cancer [77] to exposures such as environmental pollutants and disinfection byproducts. To achieve this, EXPOsOMICS used three different types of cohorts, namely experimental short-term studies, mother–child cohorts, and adult long-term studies with sample sizes from 30 (TAPAS2 study) to > 500.000 participants (EPIC CVD) in the original cohorts (see Additional file 1: Table S1 for details; [98]). However, subsamples with only limited information on their selection criteria were included in EXPOsOMICS, which could raise concerns for statistical pitfalls. In a subsample of 205 participants, personal exposure monitoring was performed to assess air pollution, movement, and biological measurements (e.g. blood; [98]). EXPOsOMICS contributions to exposure science comprise, e.g. harmonized exposure assessment for air pollution variables and methodological contributions to personal exposure monitoring. At the very least this resulted in a reduction of uncertainty regarding exposure assessment at the individual level [31]. Additionally, several statistical challenges related to exposome studies were addressed and resulted in a statistical toolkit that includes solutions for, e.g., multiple testing, the interaction of exposures, and analysis techniques for multivariate data [31]. In detail, EXPOsOMICS improved the credibility of the association between air pollution and asthma onset, which previously could have been underestimated [31]. Therefore, with help of the meet-in-the-middle approach (see “Exposure analysis strategies” section), they identified oxidative stress and subsequent inflammatory responses as potential key events in the respective adverse outcome pathways. Additional metabolomic analyses further supported mechanistic understanding and thus strengthened the evidence basis on the crucial role of ultrafine particles in their relation to adverse cardiovascular health outcomes [31]. Concerning the exposure against disinfectant byproducts (DBPs) related to swimming in chlorinated pool water, EXPOsOMICS added evidence for ‘toxicity at real life levels’ [31]. The combined analysis of metabolomics, transcriptional and microRNA changes linked the exposure to DBPs to bladder cancer, thus supporting a previously established association. Moreover, as a novel finding they also linked DBP exposure to prevalence of colorectal cancer [31].

HBM4EU, which is the most recent of the described projects, has, so far, resulted in one publication that addressed breast cancer in a sample of 585 females (age 28–85 years), who were enrolled between 2007 and 2011 [94]. A further review and the projects overall ambition promise that further work will follow [1]. Ongoing and planned work strives to link human exposure to general health status by expanding chemical analyses to biospecimen available from established health cohort studies [96] and address pesticide mixture exposure and associated health effects [101].

Overall, a wide heterogeneity remains with regard to the assessed external and internal exposures and their association with various health outcomes. These associations can either include associations of health outcomes with single exposure variables, with an aggregated number of variables or using a more comprehensive exposome measure. In order to comprehend the overall progress derived from these pioneering exposome studies, all the different variables can be summarized into four broader exposure categories for which exposure-health association have been reported. We found it useful to separate the exposure categories lifestyle, air pollution, integrative exposome groups and defined chemicals. Out of the 25 publications that were identified for the EU projects in relation to health outcomes, most associations established considered only a single or few exposures, while five HELIX publications explicitly covered exposome-based groupings; namely the pregnancy exposome [2], the early-life exposome [3, 103, 106], and the urban exposome [68]. HELIX so far published on health outcomes and their relations to integrative exposome groupings and to individual chemicals, while HEALS addressed defined chemicals and single lifestyle factors (as simple correlations and not as part of a specific, more comprehensive exposome concept). EXPOsOMICS explicitly focused on the relation of air pollution as a complex exposure to health outcomes and HBM4EU aimed at the effects of individual chemicals while viewing them as proxies for complex exposures.

In sum, the results already reported are highly promising, yet, also quite diverse in terms of study design, sample size, and novelty of insights. However, as first prime examples how a deeper understanding of the chemical exposome and related health effects can be obtained they represent a big step forward for exposure and health science. The efforts were build on major collaborative efforts of various disciplines and PIs, creative ideas, and an extensive EU third-party funding. It provides ample learning opportunities for future endeavors. It would be very valuable if the current exposome cohorts would in addition report insights into major challenges they had to solve; be it on legal and ethical aspects, the design of studies and assessment methodology, or the harmonization and sharing of data.

Besides resolving the remaining conceptual challenges, a European strategy would be helpful that supports collaboration with stakeholders including regulatory and health authorities in order to implement major findings [27, 93]. Acknowledging the need for additional coordinated efforts, the EU selected nine follow-up HORIZON 2020 Exposome projects for funding beginning January 2020. It aims “to decipher the life-long impact of external and internal exposures on human health” (https://www.humanexposome.eu) under more specific settings and summarizes the efforts in a joint cluster. With such joint efforts of science and policy, we could finally get closer to the understanding of health risks due to multiple environmental factors, in order to minimize the burden of disease, derive more effective preventive strategies, and inform future policies.

### Complementing the exposome assessment

#### The exposome in relation to the chemical universe

**Fig. 2:**
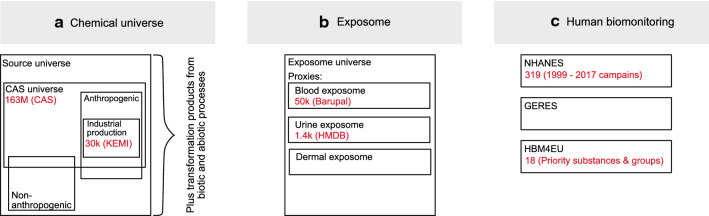
Substances in the chemical universe, the exposome, and human biomonitoring studies

An individual may be exposed to a vast number of different substances. An upper bound for this number is set by the chemical universe that summarizes all known and unknown chemicals (Fig. 2a). The Chemical Abstracts Service (CAS) Registry, as the most complete database of known chemicals, currently lists more than 163 million unique organic and inorganic chemical substances [4]. This CAS universe contains chemicals of anthropogenic and non-anthropogenic origin. However, a considerable fraction of the non-anthropogenic substances may not have been characterized so far. From an exposome perspective, many substances in the chemical universe are not relevant since they are not used or merchandised in sufficient amounts, never released to the environment, or are not of sufficient stability. A better proxy for the number of anthropogenic chemicals that are relevant for exposure considerations may be provided by the list of chemicals currently available on the market. According to the KEMI market list, which is compiled from regulatory databases, more than 30 thousand substances are expected to be available on the EU market [35]. The exposure-relevant chemical universe may however be considerably larger, due to non-anthropogenic substances and due to the products of biotic and abiotic transformation and degradation of chemicals.

The set of chemicals that comprises the chemical exposomes of individuals, i.e. the exposome’s chemical universe, is yet unknown (Fig. 2b). Furthermore, it does not only harbor chemicals on the market and their transformation products but also endogenous molecules of which the level may change in response to environmental exposure or other stress stimuli. However, several datasets have been compiled that may serve as proxies for subsets of this universe. Stephen Rappaport and colleagues compiled a set of 1561 small molecules found in human blood from the Human Metabolome Database [111] and the U.S. National Health and Nutrition Examination Survey (NHANES) [19, 79]. In a more comprehensive approach, based on text mining of PubMed abstracts and PMC full texts, Dinesh Barupal and Oliver Fiehn compiled a set of approximately 50 thousand chemicals, which they provide in a blood exposome database [8]. For urine, a similarly comprehensive analysis is missing. To obtain such an estimate of substances the Human Metabolome Database (HMDB) was queried using “Advanced search” with “Origin does not match endogenous” AND “Biolfluid matches Urine” AND “Origin is present” for non-endogenous substances found in urine. This revealed 1455 unique substances. Other proxies, like the dermal exposome obtained using skin wipes or passive samplers have been used repeatedly in exposome studies and may provide important contributions to the exposome universe (e.g. [42]. However, these initiatives on dermal exposure so far lack a broader chemical characterization.

In contrast to the list of substances included in the exposome universe, the number of chemicals included in human biomonitoring projects is minute (Fig. 2c). NHANES, as the—to our knowledge—most comprehensive monitoring program investigated 319 substances in blood and/or urine, while HBM4EU has prioritized about 230 individual compounds, for which further information should be gathered in the project or in future human biomonitoring activities. Within HBM4EU steps are undertaken to increase the number of analyzed compounds based on the development and harmonization of screening approaches [75] and the compilation as a database of suspect chemicals potentially relevant for human biomonitoring comprising 66,000 parent compounds and more than 300,000 in silico predicted metabolites [61]. Subsets of this database will be used to screen samples from cohort studies for these emerging contaminants to obtain a more comprehensive picture of chemical exposure and to prioritize compounds for further development of targeted HBM methods.

#### Complementing exposome characterization using in vitro assays

A potentially complementary approach to more comprehensively assess internal exposure would use in vitro bioassays. Here, chemicals in the extract from biosamples are quantified through detections of mixture effects for selected, typically health-relevant biological responses [38]. This approach has not been used in the exposome studies referred before.

Early work often targeted dioxin-like POPs, which act via activation of the arylhydrocarbon receptor (AhR) [23]. There are many reporter gene assays available for the AhR and some, such as the AhR-CALUX have specifically been adapted for blood analysis [65]. Environmental and blood levels of POPs could be associated to AhR activity [59]. If no acid clean-up is performed on blood extracts, naturally occurring AhR agonists cause the majority of the effect in AhR-CALUX while the effects were dependent on the diet [20]. These assays have been widely used for the analysis of tissues [110]. AhR activity in plasma has also been associated with diverse adverse outcomes.

Many environmental pollutants may cause endocrine disruption. Estrogenic activity in serum was detected as early as 1997 in human serum [90]. These early studies focused on persistent chemicals, so blood samples were solvent-extracted and processed using a sulfuric acid clean-up that digested the lipids but also non-persistent chemicals. More recent studies tried to identify xeno-estrogenic chemicals in serum with bioassays while differentiating them from endogenous hormones [12]. This method was applied to demonstrate the estrogenic effects in serum from pregnant women, which were found associated with levels of perfluorinated compounds in these samples [13]. Estrogenic activity and AhR activity was also often associated with high levels of other chemicals detected in biomonitoring [38].

Activation of the androgen receptor has been used for doping tests in urine samples to detect steroid abuse [47]. The thyroid hormone system is yet another important nuclear receptor affected by environmental pollutants [16] and binding to transthyretin has been used to identify activity in extracts from organism samples, here polar bear plasma [88].

More recently the peroxisome proliferator activated receptor has become of interest because its activation is related to lipid metabolism [41] and chemicals that activate peroxisome proliferator-activated receptor (PPAR) may be considered obesogens [48]. Recently, a serum PPAR activity assay has been developed that quantifies total PPAR ligand activity in serum [28]. Additional nuclear receptors may be involved in metabolic disruption and many of these are accessible via in vitro testing [51].

With effect-directed analysis, the mixture in an extract can be separated and stepwise bioactive chemicals and mixtures can be identified. This approach is quite popular in water and sediment quality assessment [15] but has also been used to identify estrogenic chemicals in adipose tissue [33] or thyroid activity in plasma of polar bears [88].

High-throughput screening of chemicals in large numbers using mechanistic in vitro cellular bioassays has transformed chemical risk assessment in the USA [53] and all these tools have potential for application in biomonitoring to complement chemical analysis. Bioanalysis, as an advantage, captures the entire mixture of bioactive compounds and not only selected target analytes. As for chemical analysis, one of the biggest challenges remains the sample preparation and extraction. The goal is to be as comprehensive in the extraction as possible to assure that less pollutants are overlooked. Less selective extraction, however, leads to coextraction of endogenous compounds and a lipid matrix that is typically present in much higher concentration than the micropollutants [79]. Clean-up methods are continuously improved and alternative methods such as polymer-based passive sampling are explored for this purpose. Ideally, the same sample preparation is used for chemical analysis and bioassays to allow a direct comparison of the mixture composition and the mixture effects.

## Conclusions and summary

To elucidate the role of a changing environment for the prevalence of diseases, the concept of the exposome was proposed. It strives to systematically account for the potentially large number of environmental factors that might impact human disease development. Four large collaborative EU projects are pioneering as proof-of-principles studies in order to identify environment-related health risks based on the exposome. They explored diverse means of describing individual exposure to a multitude of environmental stressors and in particular to a diversity of chemical substances of various origin. Furthermore, they elaborated means of associating multivariate exposure variables with health outcomes observed in various existing population-based European cohort studies.

In particular, the achieved progress comprised:

- Novel evidence that humans are simultaneously exposed to different environmental stressors including complex mixtures of chemicals;
- Novel evidence on health outcomes attributable to environmental stressors where humans are exposed as part of complex mixtures;
- Methodological advances to observe multiple exposures resolved in space and time, and link external and internal measurements on the level of individuals;
- Acknowledgement that chemical mixture exposure varies strongly in composition, and, therefore, assessment of individual exposomes seems adequate to identify and address relevant burdens of disease outcomes;
- Novel insights to identify risk drivers in complex mixtures and grouping of substances that jointly contribute to health risks by associating exposome and adverse health outcomes.

While the described EU projects have analyzed about 100 different substances or groups of chemicals, this provides neither a comprehensive picture of suspected exposures, nor a common rational for representative exposome proxy measures. Not surprisingly, substantial variation is found for different health outcomes with previously suspected associations between chemical exposure and adverse health effects being the most convincing. Clearly, the means to address mixture effects are unique.

## Research agenda

As the feasibility of the exposome concept has now been demonstrated at least for the part of chemical mixture exposures, future research could focus on more specific questions and the observational efforts to characterize human exposure accordingly. Strategically, while more specificity in research perspective is due, at the same time, collaborative efforts are timely to establish communication among the various follow-up projects at European and international level. A unique opportunity can be anticipated for the European human exposome network (https://www.humanexposome.eu/) that strives to cross-link the research projects in the exposome field that began in 2019. This should strive to enable the pooling of cohorts for an improved statistical data basis needed to associate multiple mixtures and their potential interactions with adverse outcomes of lesser frequency.

Objectives for future research more specifically, should comprise:

- To advance the systematics of chemical exposome assessment, i.e. tiered strategies to capture relevant exposure patterns are needed. These should employ research data methods to utilize different sources of available information to define target analytics for precise quantification of defined exposures and complement these with untargeted exposure screens to qualitatively detect patterns of emerging concern;
- For more aggregated exposome detection, complementary bioanalytical tools, such as in vitro assays and omics approaches, should be adopted and brought to high throughput format. This would also help to link different components of the exposome like the anthropogenic pollutants and other stressors;
- For identification of risk drivers in exposome assessment, complementary chemical non-target screening methods in conjunction with effect-directed analysis and combined effect models need to be developed;
- To make progress with causality in environment-health relationships, dedicated efforts are needed to combine cohort studies with exposome assessment, e.g. allowing longitudinal and cross-sectional analyses to capture the dynamics of individual exposomes, as well as to adequately identify vulnerable subgroups;
- To obtain the relevant sample sizes and by that sufficient statistical power, pooling of cohort data across European cohorts is inevitable; this, however, remains a major challenge as study participants need to consent to the respective use of their data, the data needs to be harmonized to become fully usable, and legal hurdles of data sharing between countries have to be overcome; in sum, such efforts call for more collaborative research efforts as well as anticipatory study designs, and consideration of adequate participant’s consent in order to address legal and ethical constraints;
- A structural objective to guarantee future and advanced data analysis is to accommodate high end means that allow for open access within the research community. In particular, it will be vital to define standards that enable coupling of diverse cohort studies to allow detection of small effect sizes for complex exposures of varying composition.

## Supplementary information

**Additional file 1: Table S1.** Characteristics of the used cohort studies including study design, sample size, age, time of enrolment, and gender ratio.

## Data Availability

Not applicable.

## Abbreviations

AhR: Arylhydrocarbon receptor
AOP: Adverse outcome pathways
CAS: Chemical Abstracts Service
DBP: Disinfection by-products
EU: European Union
EWAS: Environment/exposome-wide association studies
EXHES: European Exposure and Health Survey
ExWAS: Exposure Wide Association Study
FP: Framework Programme
HBM4EU: European Human Biomonitoring Initiative
HEALS: Health and Environment-wide Associations based on Large Population Surveys
HELIX: Human Early Life Exposure Project
HMDB: Human Metabolome Database
IARC: International Agency for Research on Cancer
NHANES: National Health and Nutrition Examination Survey
PAH: Polycyclic aromatic hydrocarbon
PCB: Polychloroinated byphenyls
POP: Persistent organic pollutants
PPAR: Peroxisome proliferator-activated receptors

## References

[1] Adani G, Filippini T, Wise LA, Halldorsson TI, Blaha L, Vinceti M (2020). Dietary intake of acrylamide and risk of breast, endometrial, and ovarian cancers: a systematic review and dose-response meta-analysis. Cancer Epidemiol Biomark Prev.

[2] Agier L, Basagana X, Hernandez-Ferrer C, Maitre L, Tamayo Uria I, Urquiza J, Andrusaityte S, Casas M, de Castro M, Cequier E, Chatzi L, Donaire-Gonzalez D, Giorgis-Allemand L, Gonzalez JR, Grazuleviciene R, Gutzkow KB, Haug LS, Sakhi AK, McEachan RRC, Meltzer HM, Nieuwenhuijsen M, Robinson O, Roumeliotaki T, Sunyer J, Thomsen C, Vafeiadi M, Valentin A, West J, Wright J, Siroux V, Vrijheid M, Slama R (2020). Association between the pregnancy exposome and fetal growth. Int J Epidemiol.

[3] Agier L, Basagaña X, Maitre L, Granum B, Bird PK, Casas M, Oftedal B, Wright J, Andrusaityte S, de Castro M, Cequier E, Chatzi L, Donaire-Gonzalez D, Grazuleviciene R, Haug LS, Sakhi AK, Leventakou V, McEachan R, Nieuwenhuijsen M, Petraviciene I, Robinson O, Roumeliotaki T, Sunyer J, Tamayo-Uria I, Thomsen C, Urquiza J, Valentin A, Slama R, Vrijheid M, Siroux V (2019). Early-life exposome and lung function in children in Europe: an analysis of data from the longitudinal, population-based HELIX cohort. Lancet Planet Health.

[4] American Chemical Society (2020) Chemical abstracts service registry. https://www.cas.org/support/documentation/chemical-substances. Accessed 28 July 2020

[5] Andra SS, Austin C, Arora M (2016). The tooth exposome in children's health research. Curr Opin Pediatr.

[6] Balshaw DM, Collman GW, Gray KA, Thompson CL (2017). The children's health exposure analysis resource: enabling research into the environmental influences on children's health outcomes. Curr Opin Pediatr.

[7] Barrera-Gomez J, Agier L, Portengen L, Chadeau-Hyam M, Giorgis-Allemand L, Siroux V, Robinson O, Vlaanderen J, Gonzalez JR, Nieuwenhuijsen M, Vineis P, Vrijheid M, Vermeulen R, Slama R, Basagana X (2017). A systematic comparison of statistical methods to detect interactions in exposome-health associations. Environ Health.

[8] Barupal DK, Fiehn O (2019). Generating the blood exposome database using a comprehensive text mining and database fusion approach. Environ Health Perspect.

[9] Beelen R, Hoek G, Raaschou-Nielsen O, Stafoggia M, Andersen ZJ, Weinmayr G, Hoffmann B, Wolf K, Samoli E, Fischer PH, Nieuwenhuijsen MJ, Xun WW, Katsouyanni K, Dimakopoulou K, Marcon A, Vartiainen E, Lanki T, Yli-Tuomi T, Oftedal B, Schwarze PE, Nafstad P, De Faire U, Pedersen NL, Ostenson CG, Fratiglioni L, Penell J, Korek M, Pershagen G, Eriksen KT, Overvad K, Sorensen M, Eeftens M, Peeters PH, Meliefste K, Wang M, Bueno-de-Mesquita HB, Sugiri D, Kramer U, Heinrich J, de Hoogh K, Key T, Peters A, Hampel R, Concin H, Nagel G, Jaensch A, Ineichen A, Tsai MY, Schaffner E, Probst-Hensch NM, Schindler C, Ragettli MS, Vilier A, Clavel-Chapelon F, Declercq C, Ricceri F, Sacerdote C, Galassi C, Migliore E, Ranzi A, Cesaroni G, Badaloni C, Forastiere F, Katsoulis M, Trichopoulou A, Keuken M, Jedynska A, Kooter IM, Kukkonen J, Sokhi RS, Vineis P, Brunekreef B (2015). Natural-cause mortality and long-term exposure to particle components: an analysis of 19 European cohorts within the multi-center ESCAPE project. Environ Health Perspect.

[10] Beelen R, Stafoggia M, Raaschou-Nielsen O, Andersen ZJ, Xun WW, Katsouyanni K, Dimakopoulou K, Brunekreef B, Weinmayr G, Hoffmann B, Wolf K, Samoli E, Houthuijs D, Nieuwenhuijsen M, Oudin A, Forsberg B, Olsson D, Salomaa V, Lanki T, Yli-Tuomi T, Oftedal B, Aamodt G, Nafstad P, De Faire U, Pedersen NL, Ostenson CG, Fratiglioni L, Penell J, Korek M, Pyko A, Eriksen KT, Tjonneland A, Becker T, Eeftens M, Bots M, Meliefste K, Wang M, Bueno-de-Mesquita B, Sugiri D, Kramer U, Heinrich J, de Hoogh K, Key T, Peters A, Cyrys J, Concin H, Nagel G, Ineichen A, Schaffner E, Probst-Hensch N, Dratva J, Ducret-Stich R, Vilier A, Clavel-Chapelon F, Stempfelet M, Grioni S, Krogh V, Tsai MY, Marcon A, Ricceri F, Sacerdote C, Galassi C, Migliore E, Ranzi A, Cesaroni G, Badaloni C, Forastiere F, Tamayo I, Amiano P, Dorronsoro M, Katsoulis M, Trichopoulou A, Vineis P, Hoek G (2014). Long-term exposure to air pollution and cardiovascular mortality: an analysis of 22 European cohorts. Epidemiology.

[11] Birks L, Casas M, Garcia AM, Alexander J, Barros H, Bergstrom A, Bonde JP, Burdorf A, Costet N, Danileviciute A, Eggesbo M, Fernandez MF, Gonzalez-Galarzo MC, Regina G, Hanke W, Jaddoe V, Kogevinas M, Kull I, Lertxundi A, Melaki V, Andersen AN, Olea N, Polanska K, Rusconi F, Santa-Marina L, Santos AC, Vrijkotte T, Zugna D, Nieuwenhuijsen M, Cordier S, Vrijheid M (2016). Occupational exposure to endocrine-disrupting chemicals and birth weight and length of gestation: a European meta-analysis. Environ Health Perspect.

[12] Bjerregaard-Olesen C, Bossi R, Bech BH, Bonefeld-Jorgensen EC (2015). Extraction of perfluorinated alkyl acids from human serum for determination of the combined xenoestrogenic transactivity: a method development. Chemosphere.

[13] Bjerregaard-Olesen C, Ghisari M, Bonefeld-Jorgensen EC (2016). Activation of the estrogen receptor by human serum extracts containing mixtures of perfluorinated alkyl acids from pregnant women. Environ Res.

[14] Bopp SK, Barouki R, Brack W, Dalla Costa S, Dorne JCM, Drakvik PE, Faust M, Karjalainen TK, Kephalopoulos S, van Klaveren J, Kolossa-Gehring M, Kortenkamp A, Lebret E, Lettieri T, Norager S, Ruegg J, Tarazona JV, Trier X, van de Water B, van Gils J, Bergman A (2018). Current EU research activities on combined exposure to multiple chemicals. Environ Int.

[15] Brack W (2011). Effect-directed analysis of complex environmental contamination.

[16] Brouwer A, Morse DC, Lans MC, Schuur AG, Murk AJ, Klasson-Wehler E, Bergman A, Visser TJ (1998). Interactions of persistent environmental organohalogens with the thyroid hormone system: mechanisms and possible consequences for animal and human health. Toxicol Ind Health.

[17] Buck Louis GM, Yeung E, Sundaram R, Laughon SK, Zhang C (2013). The exposome—exciting opportunities for discoveries in reproductive and perinatal epidemiology. Paediatr Perinat Epidemiol.

[18] Buekers J, David M, Koppen G, Bessems J, Scheringer M, Lebret E, Sarigiannis D, Kolossa-Gehring M, Berglund M, Schoeters G, Trier X (2018). Development of policy relevant human biomonitoring indicators for chemical exposure in the european population. Int J Environ Res Public Health.

[19] Centers for Disease Control and Prevention (2020) National Center for Health Statistics. https://www.cdc.gov/nchs/nhanes/index.htm. Accessed 28 July 2020

[20] Connor KT, Harris MA, Edwards MR, Budinsky RA, Clark GC, Chu AC, Finley BL, Rowlands JC (2008). AH receptor agonist activity in human blood measured with a cell-based bioassay: evidence for naturally occurring AH receptor ligands in vivo. J Expo Sci Environ Epidemiol.

[21] Dagnino S, Macherone A (2019). Unraveling the exposome.

[22] DeBord DG, Carreon T, Lentz TJ, Middendorf PJ, Hoover MD, Schulte PA (2016). Use of the "exposome" in the practice of epidemiology: a primer on -omic technologies. Am J Epidemiol.

[23] Denison MS, Heath-Pagliuso S (1998). The Ah receptor: a regulator of the biochemical and toxicological actions of structurally diverse chemicals. Bull Environ Contam Toxicol.

[24] Dennis KK, Marder E, Balshaw DM, Cui Y, Lynes MA, Patti GJ, Rappaport SM, Shaughnessy DT, Vrijheid M, Barr DB (2017). Biomonitoring in the era of the exposome. Environ Health Perspect.

[25] Dimakopoulou K, Samoli E, Beelen R, Stafoggia M, Andersen ZJ, Hoffmann B, Fischer P, Nieuwenhuijsen M, Vineis P, Xun W, Hoek G, Raaschou-Nielsen O, Oudin A, Forsberg B, Modig L, Jousilahti P, Lanki T, Turunen A, Oftedal B, Nafstad P, Schwarze PE, Penell J, Fratiglioni L, Andersson N, Pedersen N, Korek M, De Faire U, Eriksen KT, Tjonneland A, Becker T, Wang M, Bueno-de-Mesquita B, Tsai MY, Eeftens M, Peeters PH, Meliefste K, Marcon A, Kramer U, Kuhlbusch TA, Vossoughi M, Key T, de Hoogh K, Hampel R, Peters A, Heinrich J, Weinmayr G, Concin H, Nagel G, Ineichen A, Jacquemin B, Stempfelet M, Vilier A, Ricceri F, Sacerdote C, Pedeli X, Katsoulis M, Trichopoulou A, Brunekreef B, Katsouyanni K (2014). Air pollution and nonmalignant respiratory mortality in 16 cohorts within the ESCAPE project. Am J Respir Crit Care Med.

[26] Donaire-Gonzalez D, Valentin A, van Nunen E, Curto A, Rodriguez A, Fernandez-Nieto M, Naccarati A, Tarallo S, Tsai MY, Probst-Hensch N, Vermeulen R, Hoek G, Vineis P, Gulliver J, Nieuwenhuijsen MJ (2019). ExpoApp: an integrated system to assess multiple personal environmental exposures. Environ Int.

[27] Drakvik E, Altenburger R, Aoki Y, Backhaus T, Bahadori T, Barouki R, Brack W, Cronin MTD, Demeneix B, Hougaard Bennekou S, van Klaveren J, Kneuer C, Kolossa-Gehring M, Lebret E, Posthuma L, Reiber L, Rider C, Ruegg J, Testa G, van der Burg B, van der Voet H, Warhurst AM, van de Water B, Yamazaki K, Oberg M, Bergman A (2020). Statement on advancing the assessment of chemical mixtures and their risks for human health and the environment. Environ Int.

[28] Edwards L, Watt J, Webster TF, Schlezinger JJ (2019). Assessment of total, ligand-induced peroxisome proliferator activated receptor gamma ligand activity in serum. Environ Health.

[29] EFSA EPoPPPatRP, European Food Safety Authority (EFSA) (2013). Scientific opinion on the relevance of dissimilar mode of action and its appropriate application for cumulative risk assessment of pesticides residues in food. EFSA J.

[30] Escher BI, Hackermuller J, Polte T, Scholz S, Aigner A, Altenburger R, Bohme A, Bopp SK, Brack W, Busch W, Chadeau-Hyam M, Covaci A, Eisentrager A, Galligan JJ, Garcia-Reyero N, Hartung T, Hein M, Herberth G, Jahnke A, Kleinjans J, Kluver N, Krauss M, Lamoree M, Lehmann I, Luckenbach T, Miller GW, Muller A, Phillips DH, Reemtsma T, Rolle-Kampczyk U, Schuurmann G, Schwikowski B, Tan YM, Trump S, Walter-Rohde S, Wambaugh JF (2017). From the exposome to mechanistic understanding of chemical-induced adverse effects. Environ Int.

[31] EXPOsOMICS Project Group (2017) Final report summary—EXPOsOMICS (Enhanced exposure assessment and omic profiling for high priority environmental exposures in Europe)

[32] EXPOsOMICS Project Group (2020) EXPOsOMICS web site—air pollution. http://exposomics-project.eu/our-research/air-pollution. Accessed 28 July 2020

[33] Fernandez MF, Rivas A, Olea-Serrano F, Cerrillo I, Molina-Molina JM, Araque P, Martinez-Vidal JL, Olea N (2004). Assessment of total effective xenoestrogen burden in adipose tissue and identification of chemicals responsible for the combined estrogenic effect. Anal Bioanal Chem.

[34] Fiorito G, Vlaanderen J, Polidoro S, Gulliver J, Galassi C, Ranzi A, Krogh V, Grioni S, Agnoli C, Sacerdote C, Panico S, Tsai MY, Probst-Hensch N, Hoek G, Herceg Z, Vermeulen R, Ghantous A, Vineis P, Naccarati A, for the EXPOsOMICS Consortium (2018). Oxidative stress and inflammation mediate the effect of air pollution on cardio- and cerebrovascular disease: a prospective study in nonsmokers. Environ Mol Mutagen.

[35] Fischer S (2017) S17 | KEMIMARKET | KEMI Market List. NORMAN-SLE-S17.0.1.4 edn. 10.5281/zenodo.3959394

[36] Fraser A, Macdonald-Wallis C, Tilling K, Boyd A, Golding J, Davey Smith G, Henderson J, Macleod J, Molloy L, Ness A, Ring S, Nelson SM, Lawlor DA (2013). Cohort profile: the avon longitudinal study of parents and children: ALSPAC mothers cohort. Int J Epidemiol.

[37] Fuks KB, Weinmayr G, Foraster M, Dratva J, Hampel R, Houthuijs D, Oftedal B, Oudin A, Panasevich S, Penell J, Sommar JN, Sorensen M, Tiittanen P, Wolf K, Xun WW, Aguilera I, Basagana X, Beelen R, Bots ML, Brunekreef B, Bueno-de-Mesquita HB, Caracciolo B, Cirach M, de Faire U, de Nazelle A, Eeftens M, Elosua R, Erbel R, Forsberg B, Fratiglioni L, Gaspoz JM, Hilding A, Jula A, Korek M, Kramer U, Kunzli N, Lanki T, Leander K, Magnusson PK, Marrugat J, Nieuwenhuijsen MJ, Ostenson CG, Pedersen NL, Pershagen G, Phuleria HC, Probst-Hensch NM, Raaschou-Nielsen O, Schaffner E, Schikowski T, Schindler C, Schwarze PE, Sogaard AJ, Sugiri D, Swart WJ, Tsai MY, Turunen AW, Vineis P, Peters A, Hoffmann B (2014). Arterial blood pressure and long-term exposure to traffic-related air pollution: an analysis in the European study of cohorts for air pollution effects (ESCAPE). Environ Health Perspect.

[38] Ghisari M, Kruger T, Long M, Bonefeld-Jorgensen EC, Knudsen LE, Merlo DF (2012). Biomarkers of effects on hormone functions. Biomarkers and human biomonitoring: Vol. 2: selected biomarkers of current interest.

[39] Gillman MW, Blaisdell CJ (2018). Environmental influences on child health outcomes, a research program of the National Institutes of Health. Curr Opin Pediatr.

[40] Gromadzinska J, Polanska K, Kozlowska L, Mikolajewska K, Stelmach I, Jerzynska J, Stelmach W, Grzesiak M, Hanke W, Wasowicz W (2018). Vitamins A and E during pregnancy and allergy symptoms in an early childhood-lack of association with tobacco smoke exposure. Int J Environ Res Public Health.

[41] Grun F, Blumberg B (2007). Perturbed nuclear receptor signaling by environmental obesogens as emerging factors in the obesity crisis. Rev Endocr Metab Disord.

[42] Hammel SC, Hoffman K, Phillips AL, Levasseur JL, Lorenzo AM, Webster TF, Stapleton HM (2020). Comparing the use of silicone wristbands, hand wipes, and dust to evaluate children's exposure to flame retardants and plasticizers. Environ Sci Technol.

[43] Haug LS, Sakhi AK, Cequier E, Casas M, Maitre L, Basagana X, Andrusaityte S, Chalkiadaki G, Chatzi L, Coen M, de Bont J, Dedele A, Ferrand J, Grazuleviciene R, Gonzalez JR, Gutzkow KB, Keun H, McEachan R, Meltzer HM, Petraviciene I, Robinson O, Saulnier PJ, Slama R, Sunyer J, Urquiza J, Vafeiadi M, Wright J, Vrijheid M, Thomsen C (2018). In-utero and childhood chemical exposome in six European mother–child cohorts. Environ Int.

[44] HEALS Project Group (2013) Scientific contributions of the HEALS project. FP7-ENV-2013-603946. zenodo.org. https://zenodo.org/communities/heals/?page=1&size=20. Accessed 08 Sept 2020

[45] HEALS Project Group (2018) Periodic report summary 3—HEALS (health and environment-wide associations based on large population Surveys). https://cordis.europa.eu/project/id/603946/reporting

[46] HELIX Project Group (2018) Final report summary—HELIX (the human early-life exposome—novel tools for integrating early-life environmental exposures and child health across Europe). https://cordis.europa.eu/project/id/308333/reporting

[47] Houtman CJ, Sterk SS, van de Heijning MP, Brouwer A, Stephany RW, van der Burg B, Sonneveld E (2009). Detection of anabolic androgenic steroid abuse in doping control using mammalian reporter gene bioassays. Anal Chim Acta.

[48] Janesick A, Blumberg B (2011). Minireview: PPARγ as the target of obesogens. J Steroid Biochem Mol Biol.

[49] Janssen BG, Madlhoum N, Gyselaers W, Bijnens E, Clemente DB, Cox B, Hogervorst J, Luyten L, Martens DS, Peusens M, Plusquin M, Provost EB, Roels HA, Saenen ND, Tsamou M, Vriens A, Winckelmans E, Vrijens K, Nawrot TS (2017). Cohort profile: the ENVIRonmental influenceONearly AGEing (ENVIRONAGE): a birth cohort study. Int J Epidemiol.

[50] Kampinga MA, Vlaar PJ, Fokkema M, Gu YL, Zijlstra F (2009). Thrombus aspiration during percutaneous coronary intervention in acute non-ST-elevation myocardial infarction study (TAPAS II)-study design. Neth Heart J.

[51] Kassotis CD, Stapleton HM (2019). Endocrine-mediated mechanisms of metabolic disruption and new approaches to examine the public health threat. Front Endocrinol.

[52] Knudsen AS, Long M, Pedersen HS, Bonefeld-Jorgensen EC (2018). Persistent organic pollutants and haematological markers in Greenlandic pregnant women: the ACCEPT sub-study. Int J Circumpolar Health.

[53] Krewski D, Andersen ME, Tyshenko MG, Krishnan K, Hartung T, Boekelheide K, Wambaugh JF, Jones D, Whelan M, Thomas R, Yauk C, Barton-Maclaren T, Cote I (2020). Toxicity testing in the 21st century: progress in the past decade and future perspectives. Arch Toxicol.

[54] Landrigan PJ, Fuller R, Acosta NJR, Adeyi O, Arnold R, Basu N, Baldé AB, Bertollini R, Bose-O'Reilly S, Boufford JI, Breysse PN, Chiles T, Mahidol C, Coll-Seck AM, Cropper ML, Fobil J, Fuster V, Greenstone M, Haines A, Hanrahan D, Hunter D, Khare M, Krupnick A, Lanphear B, Lohani B, Martin K, Mathiasen KV, McTeer MA, Murray CJL, Ndahimananjara JD, Perera F, Potočnik J, Preker AS, Ramesh J, Rockström J, Salinas C, Samson LD, Sandilya K, Sly PD, Smith KR, Steiner A, Stewart RB, Suk WA, van Schayck OCP, Yadama GN, Yumkella K, Zhong M (2018). The lancet commission on pollution and health. Lancet.

[55] Lazarevic N, Barnett AG, Sly PD, Knibbs LD (2019). Statistical methodology in studies of prenatal exposure to mixtures of endocrine-disrupting chemicals: a review of existing approaches and new alternatives. Environ Health Perspect.

[56] Lewis RM (2013). The placental exposome: placental determinants of fetal adiposity and postnatal body composition. Ann Nutr Metab.

[57] Lim SS, Vos T, Flaxman AD, Danaei G, Shibuya K, Adair-Rohani H, AlMazroa MA, Amann M, Anderson HR, Andrews KG, Aryee M, Atkinson C, Bacchus LJ, Bahalim AN, Balakrishnan K, Balmes J, Barker-Collo S, Baxter A, Bell ML, Blore JD, Blyth F, Bonner C, Borges G, Bourne R, Boussinesq M, Brauer M, Brooks P, Bruce NG, Brunekreef B, Bryan-Hancock C, Bucello C, Buchbinder R, Bull F, Burnett RT, Byers TE, Calabria B, Carapetis J, Carnahan E, Chafe Z, Charlson F, Chen H, Chen JS, Cheng AT-A, Child JC, Cohen A, Colson KE, Cowie BC, Darby S, Darling S, Davis A, Degenhardt L, Dentener F, Des Jarlais DC, Devries K, Dherani M, Ding EL, Dorsey ER, Driscoll T, Edmond K, Ali SE, Engell RE, Erwin PJ, Fahimi S, Falder G, Farzadfar F, Ferrari A, Finucane MM, Flaxman S, Fowkes FGR, Freedman G, Freeman MK, Gakidou E, Ghosh S, Giovannucci E, Gmel G, Graham K, Grainger R, Grant B, Gunnell D, Gutierrez HR, Hall W, Hoek HW, Hogan A, Hosgood HD, Hoy D, Hu H, Hubbell BJ, Hutchings SJ, Ibeanusi SE, Jacklyn GL, Jasrasaria R, Jonas JB, Kan H, Kanis JA, Kassebaum N, Kawakami N, Khang Y-H, Khatibzadeh S, Khoo J-P, Kok C, Laden F, Lalloo R, Lan Q, Lathlean T, Leasher JL, Leigh J, Li Y, Lin JK, Lipshultz SE, London S, Lozano R, Lu Y, Mak J, Malekzadeh R, Mallinger L, Marcenes W, March L, Marks R, Martin R, McGale P, McGrath J, Mehta S, Memish ZA, Mensah GA, Merriman TR, Micha R, Michaud C, Mishra V, Hanafiah KM, Mokdad AA, Morawska L, Mozaffarian D, Murphy T, Naghavi M, Neal B, Nelson PK, Nolla JM, Norman R, Olives C, Omer SB, Orchard J, Osborne R, Ostro B, Page A, Pandey KD, Parry CDH, Passmore E, Patra J, Pearce N, Pelizzari PM, Petzold M, Phillips MR, Pope D, Pope CA, Powles J, Rao M, Razavi H, Rehfuess EA, Rehm JT, Ritz B, Rivara FP, Roberts T, Robinson C, Rodriguez-Portales JA, Romieu I, Room R, Rosenfeld LC, Roy A, Rushton L, Salomon JA, Sampson U, Sanchez-Riera L, Sanman E, Sapkota A, Seedat S, Shi P, Shield K, Shivakoti R, Singh GM, Sleet DA, Smith E, Smith KR, Stapelberg NJC, Steenland K, Stöckl H, Stovner LJ, Straif K, Straney L, Thurston GD, Tran JH, Van Dingenen R, van Donkelaar A, Veerman JL, Vijayakumar L, Weintraub R, Weissman MM, White RA, Whiteford H, Wiersma ST, Wilkinson JD, Williams HC, Williams W, Wilson N, Woolf AD, Yip P, Zielinski JM, Lopez AD, Murray CJL, Ezzati M (2012). A comparative risk assessment of burden of disease and injury attributable to 67 risk factors and risk factor clusters in 21 regions, 1990–2010: a systematic analysis for the global burden of disease study 2010. Lancet.

[58] Loh M, Sarigiannis D, Gotti A, Karakitsios S, Pronk A, Kuijpers E, Annesi-Maesano I, Baiz N, Madureira J, Oliveira Fernandes E, Jerrett M, Cherrie JW (2017). How sensors might help define the external exposome. Int J Environ Res Public Health.

[59] Long M, Bonefeld-Jørgensen EC (2012). Dioxin-like activity in environmental and human samples from Greenland and Denmark. Chemosphere.

[60] Maitre L, de Bont J, Casas M, Robinson O, Aasvang GM, Agier L, Andrusaityte S, Ballester F, Basagana X, Borras E, Brochot C, Bustamante M, Carracedo A, de Castro M, Dedele A, Donaire-Gonzalez D, Estivill X, Evandt J, Fossati S, Giorgis-Allemand L, Gonzalez JR, Granum B, Grazuleviciene R, Bjerve Gutzkow K, Smastuen Haug L, Hernandez-Ferrer C, Heude B, Ibarluzea J, Julvez J, Karachaliou M, Keun HC, Hjertager Krog N, Lau CE, Leventakou V, Lyon-Caen S, Manzano C, Mason D, McEachan R, Meltzer HM, Petraviciene I, Quentin J, Roumeliotaki T, Sabido E, Saulnier PJ, Siskos AP, Siroux V, Sunyer J, Tamayo I, Urquiza J, Vafeiadi M, van Gent D, Vives-Usano M, Waiblinger D, Warembourg C, Chatzi L, Coen M, van den Hazel P, Nieuwenhuijsen MJ, Slama R, Thomsen C, Wright J, Vrijheid M (2018). Human early life exposome (HELIX) study: a European population-based exposome cohort. BMJ Open.

[61] Meijer J, Lamoree M, Hamers T, Antignac J-P, Hutinet S, Debrauwer L, Covaci A, Huber C, Krauss M, Walker DI, Schymanski EL, Vermeulen R, Vlaanderen J (2020) A suspect screening database for chemicals of emerging concern in exposome research. Environ Int (**under revision**) 10.1016/j.envint.2021.106511 33773387

[62] Menni C, Migaud M, Kastenmuller G, Pallister T, Zierer J, Peters A, Mohney RP, Spector TD, Bagnardi V, Gieger C, Moore SC, Valdes AM (2017). Metabolomic profiling of long-term weight change: role of oxidative stress and urate levels in weight gain. Obesity.

[63] Miller GW, Jones DP (2014). The nature of nurture: refining the definition of the exposome. Toxicol Sci.

[64] Montazeri P, Thomsen C, Casas M, de Bont J, Haug LS, Maitre L, Papadopoulou E, Sakhi AK, Slama R, Saulnier PJ, Urquiza J, Grazuleviciene R, Andrusaityte S, McEachan R, Wright J, Chatzi L, Basagana X, Vrijheid M (2019). Socioeconomic position and exposure to multiple environmental chemical contaminants in six European mother-child cohorts. Int J Hyg Environ Health.

[65] Murk AJ, Leonards PEG, Bulder AS, Jonas AS, Rozemeijer MJC, Denison MS, Koeman JH, Brouwer A (1997). The calux (chemical-activated luciferase expression) assay adapted and validated for measuring TCDD equivalents in blood plasma. Environ Toxicol Chem.

[66] Neveu VMA, Rouaix H, Wedekind R, Pon A, Knox C, Wishart DS, Scalbert A (2017). Exposome-explorer: a manually-curated database on biomarkers of exposure to dietary and environmental factors. Nucleic Acids Res.

[67] Neveu VNG, Salek RM, Wishart DS, Scalbert A (2019). Exposome-Explorer 2.0: an update incorporating candidate dietary biomarkers and dietary associations with cancer risk. Nucleic Acids Res.

[68] Nieuwenhuijsen MJ, Agier L, Basagana X, Urquiza J, Tamayo-Uria I, Giorgis-Allemand L, Robinson O, Siroux V, Maitre L, de Castro M, Valentin A, Donaire D, Dadvand P, Aasvang GM, Krog NH, Schwarze PE, Chatzi L, Grazuleviciene R, Andrusaityte S, Dedele A, McEachan R, Wright J, West J, Ibarluzea J, Ballester F, Vrijheid M, Slama R (2019). Influence of the urban exposome on birth weight. Environ Health Perspect.

[69] Ougier E, Lecoq P, Rouselle C, Ormsby J-N (2018) HBM4EU deliverable 4.5: second list of HBM4EU priority substances and Chemical Substance Group Leaders for 2019–2021

[70] Polanska K, Hanke W, Krol A, Gromadzinska J, Kuras R, Janasik B, Wasowicz W, Mirabella F, Chiarotti F, Calamandrei G (2017). Micronutrients during pregnancy and child psychomotor development: opposite effects of Zinc and Selenium. Environ Res.

[71] Polanska K, Hanke W, Pawlas N, Wesolowska E, Jankowska A, Jagodic M, Mazej D, Dominowska J, Grzesiak M, Mirabella F, Chiarotti F, Calamandrei G (2018). Sex-dependent impact of low-level lead exposure during prenatal period on child psychomotor functions. Int J Environ Res Public Health.

[72] Polanska K, Krol A, Merecz-Kot D, Ligocka D, Mikolajewska K, Mirabella F, Chiarotti F, Calamandrei G, Hanke W (2017). Environmental tobacco smoke exposure during pregnancy and child neurodevelopment. Int J Environ Res Public Health.

[73] Polanska K, Krol A, Merecz-Kot D, Jurewicz J, Makowiec-Dabrowska T, Chiarotti F, Calamandrei G, Hanke W (2017). Maternal stress during pregnancy and neurodevelopmental outcomes of children during the first 2 years of life. J Paediatr Child Health.

[74] Polanska K, Krol A, Sobala W, Gromadzinska J, Brodzka R, Calamandrei G, Chiarotti F, Wasowicz W, Hanke W (2016). Selenium status during pregnancy and child psychomotor development-Polish mother and child cohort study. Pediatr Res.

[75] Pourchet M, Debrauwer L, Klanova J, Price EJ, Covaci A, Caballero-Casero N, Oberacher H, Lamoree M, Damont A, Fenaille F, Vlaanderen J, Meijer J, Krauss M, Sarigiannis D, Barouki R, Le Bizec B, Antignac JP (2020). Suspect and non-targeted screening of chemicals of emerging concern for human biomonitoring, environmental health studies and support to risk assessment: from promises to challenges and harmonisation issues. Environ Int.

[76] Prpić I, Milardović A, Vlašić-Cicvarić I, Špiric Z, Nišević JR, Vukelić P, Tratnik JS, Mazej D, Horvat MJ (2017). Prenatal exposure to low-level methylmercury alters the child's fine motor skills at the age of 18 months. Environ Res.

[77] Raaschou-Nielsen O, Andersen ZJ, Beelen R, Samoli E, Stafoggia M, Weinmayr G, Hoffmann B, Fischer P, Nieuwenhuijsen MJ, Brunekreef B, Xun WW, Katsouyanni K, Dimakopoulou K, Sommar J, Forsberg B, Modig L, Oudin A, Oftedal B, Schwarze PE, Nafstad P, De Faire U, Pedersen NL, Östenson C-G, Fratiglioni L, Penell J, Korek M, Pershagen G, Eriksen KT, Sørensen M, Tjønneland A, Ellermann T, Eeftens M, Peeters PH, Meliefste K, Wang M, Bueno-de-Mesquita B, Key TJ, de Hoogh K, Concin H, Nagel G, Vilier A, Grioni S, Krogh V, Tsai M-Y, Ricceri F, Sacerdote C, Galassi C, Migliore E, Ranzi A, Cesaroni G, Badaloni C, Forastiere F, Tamayo I, Amiano P, Dorronsoro M, Trichopoulou A, Bamia C, Vineis P, Hoek G (2013). Air pollution and lung cancer incidence in 17 European cohorts: prospective analyses from the European study of cohorts for air pollution effects (ESCAPE). Lancet Oncol.

[78] Rappaport SM (2011). Implications of the exposome for exposure science. J Expo Sci Environ Epidemiol.

[79] Rappaport SM, Barupal DK, Wishart D, Vineis P, Scalbert A (2014). The blood exposome and its role in discovering causes of disease. Environ Health Perspect.

[80] Rappaport SM, Li H, Grigoryan H, Funk WE, Williams ER (2012). Adductomics: characterizing exposures to reactive electrophiles. Toxicol Lett.

[81] Rappaport SM, Smith MT (2010). Environment and disease risks. Science.

[82] Robinson O, Vrijheid M (2015). The pregnancy exposome. Curr Environ Health Rep.

[83] Rogler G, Vavricka S (2015). Exposome in IBD: recent insights in environmental factors that influence the onset and course of IBD. Inflamm Bowel Dis.

[84] Santonen T, Heinälä M, Bessems J, Buekers J, Cornelis C, Vermeire T, Woutersen M, van Engelen J, Borges T, Rousselle C, Ougier E, Louro H, Alvito P, Martins C, Assunção R, Silva MJ, Krul L, Pronk A, Schaddelee-Scholten B, Stierum R, Gonzalez MC, de Alba M, Díaz G, Castaño A, Viegas S, Humar-Juric T, Kononenko L, Abraham K, Vinggaard AM (2017) HBM4EU deliverable 5.1: human biomonitoring in risk assessment: analysis of the current practice and 1st examples on the use of HBM in risk assessments of HBM4EU priority chemicals

[85] Santos S, Maitre L, Warembourg C, Agier L, Richiardi L, Basagana X, Vrijheid M (2020). Applying the exposome concept in birth cohort research: a review of statistical approaches. Eur J Epidemiol.

[86] Schoeters G, Tschersich C, Barouki R, Uhl M, Klánová J, Horvat M, Alimonti A, Sarigiannis D, Santonen T, Lebret E (2017) HBM4EU deliverable 4.2: scoping documents on HBM4EU priority substances for 2018–2017

[87] Sharma RP, Schuhmacher M, Kumar V (2018). The development of a pregnancy PBPK model for bisphenol A and its evaluation with the available biomonitoring data. Sci Total Environ.

[88] Simon E, van Velzen M, Brandsma SH, Lie E, Løken K, de Boer J, Bytingsvik J, Jenssen BM, Aars J, Hamers T, Lamoree MH (2013). Effect-directed analysis to explore the polar bear exposome: identification of thyroid hormone disrupting compounds in plasma. Environ Sci Technol.

[89] Snoj Tratnik J, Falnoga I, Trdin A, Mazej D, Fajon V, Miklavcic A, Kobal AB, Osredkar J, Sesek Briski A, Krsnik M, Neubauer D, Kodric J, Stropnik S, Gosar D, Lesnik Musek P, Marc J, Jurkovic Mlakar S, Petrovic O, Vlasic-Cicvaric I, Prpic I, Milardovic A, Radic Nisevic J, Vukovic D, Fisic E, Spiric Z, Horvat M (2017). Prenatal mercury exposure, neurodevelopment and apolipoprotein E genetic polymorphism. Environ Res.

[90] Soto AM, Fernandez MF, Luizzi MF, Oles Karasko AS, Sonnenschein C (1997). Developing a marker of exposure to xenoestrogen mixtures in human serum. Environ Health Perspect.

[91] Stafoggia M, Cesaroni G, Peters A, Andersen ZJ, Badaloni C, Beelen R, Caracciolo B, Cyrys J, de Faire U, de Hoogh K, Eriksen KT, Fratiglioni L, Galassi C, Gigante B, Havulinna AS, Hennig F, Hilding A, Hoek G, Hoffmann B, Houthuijs D, Korek M, Lanki T, Leander K, Magnusson PK, Meisinger C, Migliore E, Overvad K, Ostenson CG, Pedersen NL, Pekkanen J, Penell J, Pershagen G, Pundt N, Pyko A, Raaschou-Nielsen O, Ranzi A, Ricceri F, Sacerdote C, Swart WJ, Turunen AW, Vineis P, Weimar C, Weinmayr G, Wolf K, Brunekreef B, Forastiere F (2014). Long-term exposure to ambient air pollution and incidence of cerebrovascular events: results from 11 European cohorts within the ESCAPE project. Environ Health Perspect.

[92] Steckling N, Gotti A, Bose-O'Reilly S, Chapizanis D, Costopoulou D, De Vocht F, Gari M, Grimalt JO, Heath E, Hiscock R, Jagodic M, Karakitsios SP, Kedikoglou K, Kosjek T, Leondiadis L, Maggos T, Mazej D, Polanska K, Povey A, Rovira J, Schoierer J, Schuhmacher M, Spiric Z, Stajnko A, Stierum R, Tratnik JS, Vassiliadou I, Annesi-Maesano I, Horvat M, Sarigiannis DA (2018). Biomarkers of exposure in environment-wide association studies—opportunities to decode the exposome using human biomonitoring data. Environ Res.

[93] Stingone JA, Buck Louis GM, Nakayama SF, Vermeulen RC, Kwok RK, Cui Y, Balshaw DM, Teitelbaum SL (2017). Toward greater implementation of the exposome research paradigm within environmental epidemiology. Annu Rev Public Health.

[94] Strumylaite L, Kregzdyte R, Bogusevicius A, Poskiene L, Baranauskiene D, Pranys D (2019). Cadmium exposure and risk of breast cancer by histological and tumor receptor subtype in white Caucasian Women: a hospital-based case-control study. Int J Mol Sci.

[95] Tamayo-Uria I, Maitre L, Thomsen C, Nieuwenhuijsen MJ, Chatzi L, Siroux V, Aasvang GM, Agier L, Andrusaityte S, Casas M, de Castro M, Dedele A, Haug LS, Heude B, Grazuleviciene R, Gutzkow KB, Krog NH, Mason D, McEachan RRC, Meltzer HM, Petraviciene I, Robinson O, Roumeliotaki T, Sakhi AK, Urquiza J, Vafeiadi M, Waiblinger D, Warembourg C, Wright J, Slama R, Vrijheid M, Basagana X (2019). The early-life exposome: description and patterns in six European countries. Environ Int.

[96] Tolonen H, Andersson A-M, Fiddicke U, Kold Jensen T, Májek O, Meltzer HM, Moshammer H, Paalanen L, Wennberg M, Åkesson A (2019) Selection of feasibility studies for linking HBM and health studies, and linking to administrative data sources

[97] Turner MC, Nieuwenhuijsen M, Anderson K, Balshaw D, Cui Y, Dunton G, Hoppin JA, Koutrakis P, Jerrett M (2017). Assessing the exposome with external measures: commentary on the state of the science and research recommendations. Annu Rev Public Health.

[98] Vineis P, Chadeau-Hyam M, Gmuender H, Gulliver J, Herceg Z, Kleinjans J, Kogevinas M, Kyrtopoulos S, Nieuwenhuijsen M, Phillips DH, Probst-Hensch N, Scalbert A, Vermeulen R, Wild CP, Consortium EX (2017). The exposome in practice: design of the EXPOsOMICS project. Int J Hyg Environ Health.

[99] Vineis P, van Veldhoven K, Chadeau-Hyam M, Athersuch TJ (2013). Advancing the application of omics-based biomarkers in environmental epidemiology. Environ Mol Mutagen.

[100] Vizcaino E, Grimalt JO, Glomstad B, Fernandez-Somoano A, Tardon A (2014). Gestational weight gain and exposure of newborns to persistent organic pollutants. Environ Health Perspect.

[101] Vlaanderen J, Ottenbros I, Lebret E, Bogers R, Vermeulen R, Antignac J-P, Krauss M, Debrauwer L, Oberacher H (2019) Report outline and workplan for the joint survey on HBM mixtures: an application to pesticide exposure in hotspots and control areas

[102] Vrijheid M (2014). The exposome: a new paradigm to study the impact of environment on health. Thorax.

[103] Vrijheid M, Fossati S, Maitre L, Marquez S, Roumeliotaki T, Agier L, Andrusaityte S, Cadiou S, Casas M, de Castro M, Dedele A, Donaire-Gonzalez D, Grazuleviciene R, Haug LS, McEachan R, Meltzer HM, Papadopouplou E, Robinson O, Sakhi AK, Siroux V, Sunyer J, Schwarze PE, Tamayo-Uria I, Urquiza J, Vafeiadi M, Valentin A, Warembourg C, Wright J, Nieuwenhuijsen MJ, Thomsen C, Basagana X, Slama R, Chatzi L (2020). Early-life environmental exposures and childhood obesity: an exposome-wide approach. Environ Health Perspect.

[104] Vrijheid M, Slama R, Robinson O, Chatzi L, Coen M, van den Hazel P, Thomsen C, Wright J, Athersuch TJ, Avellana N, Basagaña X, Brochot C, Bucchini L, Bustamante M, Carracedo A, Casas M, Estivill X, Fairley L, van Gent D, Gonzalez JR, Granum B, Gražulevičienė R, Gutzkow KB, Julvez J, Keun HC, Kogevinas M, McEachan RRC, Meltzer HM, Sabidó E, Schwarze PE, Siroux V, Sunyer J, Want EJ, Zeman F, Nieuwenhuijsen MJ (2014). The human early-life exposome (HELIX): project rationale and design. Environ Health Perspect.

[105] Warembourg C, Basagana X, Seminati C, de Bont J, Granum B, Lyon-Caen S, Manzano-Salgado CB, Pin I, Sakhi AK, Siroux V, Slama R, Urquiza J, Vrijheid M, Thomsen C, Casas M (2019). Exposure to phthalate metabolites, phenols and organophosphate pesticide metabolites and blood pressure during pregnancy. Int J Hyg Environ Health.

[106] Warembourg C, Maitre L, Tamayo-Uria I, Fossati S, Roumeliotaki T, Aasvang GM, Andrusaityte S, Casas M, Cequier E, Chatzi L, Dedele A, Gonzalez JR, Grazuleviciene R, Haug LS, Hernandez-Ferrer C, Heude B, Karachaliou M, Krog NH, McEachan R, Nieuwenhuijsen M, Petraviciene I, Quentin J, Robinson O, Sakhi AK, Slama R, Thomsen C, Urquiza J, Vafeiadi M, West J, Wright J, Vrijheid M, Basagana X (2019). Early-life environmental exposures and blood pressure in children. J Am Coll Cardiol.

[107] Wielsoe M, Tarantini L, Bollati V, Long M, Bonefeld-Jorgensen EC (2020). DNA methylation level in blood and relations to breast cancer, risk factors and environmental exposure in Greenlandic Inuit women. Basic Clin Pharmacol Toxicol.

[108] Wild CP (2005). Complementing the genome with an "exposome": the outstanding challenge of environmental exposure measurement in molecular epidemiology. Cancer Epidemiol Biomark Prev.

[109] Wild CP (2012). The exposome: from concept to utility. Int J Epidemiol.

[110] Windal I, Van Wouwe N, Eppe G, Xhrouet C, Debacker V, Baeyens W, De Pauw E, Goeyens L (2005). Validation and interpretation of CALUX as a tool for the estimation of dioxin-like activity in marine biological matrixes. Environ Sci Technol.

[111] Wishart DS, Feunang YD, Marcu A, Guo AC, Liang K, Vazquez-Fresno R, Sajed T, Johnson D, Li C, Karu N, Sayeeda Z, Lo E, Assempour N, Berjanskii M, Singhal S, Arndt D, Liang Y, Badran H, Grant J, Serra-Cayuela A, Liu Y, Mandal R, Neveu V, Pon A, Knox C, Wilson M, Manach C, Scalbert A (2018). HMDB 4.0: the human metabolome database for 2018. Nucleic Acids Res.

[112] Wright J, Small N, Raynor P, Tuffnell D, Bhopal R, Cameron N, Fairley L, Lawlor DA, Parslow R, Petherick ES, Pickett KE, Waiblinger D, West J, Born in Bradford Scientific Collaborators Group (2013). Cohort Profile: The Born in Bradford multi-ethnic family cohort study. Int J Epidemiol.

