# Supplementary material for: Unravelling the chemical exposome in cohort studies: routes explored and steps to become comprehensive
Source: Environ Sci Eur. 2021 Feb 11;33(1):17. doi: 10.1186/s12302-020-00444-0 (PMC7877320; doi:10.1186/s12302-020-00444-0)
Supplement: Supplementary file 1 — Additional file 1: Table S1. Characteristics of the used cohort studies including study design, sample size, age, time of enrolment, and gender ratio. [file 12302_2020_444_MOESM1_ESM.docx]

**Additional file**

**Table S1.** Characteristics of the used cohort studies including study design, sample size, age, time of enrolment, and gender ratio.

*sample size for HELIX as described in Vrijheid et al. 2014, **sample size within HEALS as described in respective references.

Abbreviations: ABCD: Amsterdam Born Children and their Development, ALSPAC: The Avon Longitudinal Study of Parents and Children, BAMSE: Swedish abbreviation for Childre, Allergy, Milieu, Stockholm, Epidemiology, BIB: Born in Bradford, DNBC: Danish National Birth Cohort, EDEN: Study on the pre- and early postnatal determinants of child health and development, ENVIRonAGE: ENVIRonmental influence ON AGEing in early life, EPIC CVD: European Prospective Investigation Into Cancer - Cardiovascular Diseases, INMA: Infancia y Medio Ambiente (environment and childhood), KANC: Kaunas Cohort, KORA: Cooperative Health Research in the Region Augsburg, n.a.: not available, MCC: Multi Cancer Control Study Spain, MoBa: Norwegian Mother, Father and Child Cohort Study, NINFEA: Nascita ed INFancia: gli Effetti dell'Ambiente), PELAGIE: Perturbateurs Endocriniens : Étude Longitudinale sur les Anomalies de la Grossesse, l’Infertilité et l’Enfance, PHIME: Public health impact of long-term, low-level mixed element exposure in susceptible population strata, PLCO: Prostate, Lung, Colorectal, Ovarian, REPRO_PL: Polish Mother and Child Cohort, RHEA: The Mother-Child Cohort in Crete, Greece;

References:

Ahn J, Moore SC, Albanes D, Huang WY, Leitzmann MF, Hayes RB, Prostate LC, Ovarian Cancer Screening Trial Project T (2009) Height and risk of prostate cancer in the prostate, lung, colorectal, and ovarian cancer screening trial. Br J Cancer 101 (3):522-525. doi:10.1038/sj.bjc.6605159

Barbone F, Rosolen V, Mariuz M, Parpinel M, Casetta A, Sammartano F, Ronfani L, Vecchi Brumatti L, Bin M, Castriotta L, Valent F, Little DL, Mazej D, Snoj Tratnik J, Miklavcic Visnjevec A, Sofianou K, Spiric Z, Krsnik M, Osredkar J, Neubauer D, Kodric J, Stropnik S, Prpic I, Petrovic O, Vlasic-Cicvaric I, Horvat M (2019) Prenatal mercury exposure and child neurodevelopment outcomes at 18 months: Results from the Mediterranean PHIME cohort. Int J Hyg Environ Health 222 (1):9-21. doi:10.1016/j.ijheh.2018.07.011

Birks L, Casas M, Garcia AM, Alexander J, Barros H, Bergstrom A, Bonde JP, Burdorf A, Costet N, Danileviciute A, Eggesbo M, Fernandez MF, Gonzalez-Galarzo MC, Regina G, Hanke W, Jaddoe V, Kogevinas M, Kull I, Lertxundi A, Melaki V, Andersen AN, Olea N, Polanska K, Rusconi F, Santa-Marina L, Santos AC, Vrijkotte T, Zugna D, Nieuwenhuijsen M, Cordier S, Vrijheid M (2016) Occupational Exposure to Endocrine-Disrupting Chemicals and Birth Weight and Length of Gestation: A European Meta-Analysis. Environ Health Perspect 124 (11):1785-1793. doi:10.1289/EHP208

Boyd A, Thomas R, Hansell AL, Gulliver J, Hicks LM, Griggs R, Vande Hey J, Taylor CM, Morris T, Golding J, Doerner R, Fecht D, Henderson J, Lawlor DA, Timpson NJ, Macleod J (2019) Data Resource Profile: The ALSPAC birth cohort as a platform to study the relationship of environment and health and social factors. Int J Epidemiol 48 (4):1038-1039k. doi:10.1093/ije/dyz063

Burney PG, Luczynska C, Chinn S, Jarvis D (1994) The European Community Respiratory Health Survey. Eur Respir J 7 (5):954-960. doi:10.1183/09031936.94.07050954

Castano-Vinyals G, Aragones N, Perez-Gomez B, Martin V, Llorca J, Moreno V, Altzibar JM, Ardanaz E, de Sanjose S, Jimenez-Moleon JJ, Tardon A, Alguacil J, Peiro R, Marcos-Gragera R, Navarro C, Pollan M, Kogevinas M, Group MC-SS (2015) Population-based multicase-control study in common tumors in Spain (MCC-Spain): rationale and study design. Gac Sanit 29 (4):308-315. doi:10.1016/j.gaceta.2014.12.003

Chatzi L, Plana E, Daraki V, Karakosta P, Alegkakis D, Tsatsanis C, Kafatos A, Koutis A, Kogevinas M (2009) Metabolic Syndrome in Early Pregnancy and Risk of Preterm Birth. American Journal of Epidemiology 170 (7):829-836. doi:10.1093/aje/kwp211

Dierssen-Sotos T, Gomez-Acebo I, de Pedro M, Perez-Gomez B, Servitja S, Moreno V, Amiano P, Fernandez-Villa T, Barricarte A, Tardon A, Diaz-Santos M, Peiro-Perez R, Marcos-Gragera R, Lope V, Gracia-Lavedan E, Alonso MH, Michelena-Echeveste MJ, Garcia-Palomo A, Guevara M, Castano-Vinyals G, Aragones N, Kogevinas M, Pollan M, Llorca J (2016) Use of non-steroidal anti-inflammatory drugs and risk of breast cancer: The Spanish Multi-Case-control (MCC) study. Bmc Cancer 16. doi:ARTN 660

10.1186/s12885-016-2692-4

Drouillet P, Kaminski M, De Lauzon-Guillain B, Forhan A, Ducimetiere P, Schweitzer M, Magnin G, Goua V, Thiebaugeorges O, Charles MA (2009) Association between maternal seafood consumption before pregnancy and fetal growth: evidence for an association in overweight women. The EDEN mother-child cohort. Paediatr Perinat Epidemiol 23 (1):76-86. doi:10.1111/j.1365-3016.2008.00982.x

Farchi S, Forastiere F, Brumatti LV, Alviti S, Arnofi A, Bernardini T, Bin M, Brescianini S, Colelli V, Cotichini R, Culasso M, De Bartolo P, Felice L, Fiano V, Fioritto A, Frizzi A, Gagliardi L, Giorgi G, Grasso C, La Rosa F, Loganes C, Lorusso P, Martini V, Merletti F, Medda E, Montelatici V, Mugelli I, Narduzzi S, Nistico L, Penna L, Piscianz E, Piscicelli C, Poggesi G, Porta D, Ranieli A, Rapisardi G, Rasulo A, Richiardi L, Rusconi F, Serino L, Stazi MA, Toccaceli V, Todros T, Tognin V, Trevisan M, Valencic E, Volpi P, Ziroli V, Ronfani L, Di Lallo D (2014) Piccolipiu, a multicenter birth cohort in Italy: protocol of the study. Bmc Pediatrics 14. doi:Artn 36

10.1186/1471-2431-14-36

Font-Ribera L, Marco E, Grimalt JO, Pastor S, Marcos R, Abramsson-Zetterberg L, Pedersen M, Grummt T, Junek R, Barreiro E, Heederik D, Spithoven J, Critelli R, Naccarati A, Schmalz C, Zwiener C, Liu J, Zhang X, Mitch W, Gracia-Lavedan E, Arjona L, de Bont J, Tarès L, Vineis P, Kogevinas M, Villanueva CM (2019) Exposure to disinfection by-products in swimming pools and biomarkers of genotoxicity and respiratory damage – The PISCINA2 Study. Environment International 131. doi:10.1016/j.envint.2019.104988

Gohagan JK, Prorok PC, Hayes RB, Kramer B-S (2000) The Prostate, Lung, Colorectal and Ovarian (PLCO) Cancer Screening Trial of the National Cancer Institute: History, organization, and status. Controlled Clinical Trials 21 (6):251S-272S. doi:10.1016/s0197-2456(00)00097-0

Grazuleviciene R, Danileviciute A, Nadisauskiene R, Vencloviene J (2009) Maternal smoking, GSTM1 and GSTT1 polymorphism and susceptibility to adverse pregnancy outcomes. Int J Environ Res Public Health 6 (3):1282-1297. doi:10.3390/ijerph6031282

Gromadzinska J, Polanska K, Kozlowska L, Mikolajewska K, Stelmach I, Jerzynska J, Stelmach W, Grzesiak M, Hanke W, Wasowicz W (2018) Vitamins A and E during Pregnancy and Allergy Symptoms in an Early Childhood-Lack of Association with Tobacco Smoke Exposure. Int J Environ Res Public Health 15 (6). doi:10.3390/ijerph15061245

Guxens M, Ballester F, Espada M, Fernandez MF, Grimalt JO, Ibarluzea J, Olea N, Rebagliato M, Tardon A, Torrent M, Vioque J, Vrijheid M, Sunyer J, Project I (2012) Cohort Profile: the INMA--INfancia y Medio Ambiente--(Environment and Childhood) Project. Int J Epidemiol 41 (4):930-940. doi:10.1093/ije/dyr054

Holle R, Happich M, Lowel H, Wichmann HE, Group MKS (2005) KORA--a research platform for population based health research. Gesundheitswesen 67 Suppl 1:S19-25. doi:10.1055/s-2005-858235

Janssen BG, Madlhoum N, Gyselaers W, Bijnens E, Clemente DB, Cox B, Hogervorst J, Luyten L, Martens DS, Peusens M, Plusquin M, Provost EB, Roels HA, Saenen ND, Tsamou M, Vriens A, Winckelmans E, Vrijens K, Nawrot TS (2017) Cohort Profile: The ENVIRonmental influenceONearly AGEing (ENVIRONAGE): a birth cohort study. International Journal of Epidemiology. doi:10.1093/ije/dyw269

Kampinga MA, Vlaar PJ, Fokkema M, Gu YL, Zijlstra F (2009) Thrombus Aspiration during Percutaneous coronary intervention in Acute non-ST-elevation myocardial infarction Study (TAPAS II)-Study design. Neth Heart J 17 (11):409-413. doi:10.1007/BF03086293

Magnus P, Birke C, Vejrup K, Haugan A, Alsaker E, Daltveit AK, Handal M, Haugen M, Hoiseth G, Knudsen GP, Paltiel L, Schreuder P, Tambs K, Vold L, Stoltenberg C (2016) Cohort Profile Update: The Norwegian Mother and Child Cohort Study (MoBa). Int J Epidemiol 45 (2):382-388. doi:10.1093/ije/dyw029

Magnus P, Irgens LM, Haug K, Nystad W, Skjaerven R, Stoltenberg C (2006) Cohort profile: The Norwegian Mother and Child Cohort Study (MoBa). International Journal of Epidemiology 35 (5):1146-1150. doi:10.1093/ije/dyl170

Martin BW, AckermannLiebrich U, Leuenberger P, Kunzli N, Stutz EZ, Keller R, Zellweger JP, Wuthrich B, Monn C, Blaser K, Bolognini G, Bongard JP, Brandli O, Braun P, Defila C, Domenighetti G, Grize L, Karrer W, KellerWossidlo H, Medici TC, Peeters A, Perruchoud AP, Schindler C, Schoeni MH, Schwartz J, Solari G, Tschopp JM, Villiger B (1997) SAPALDIA: Methods and participation in the cross-sectional part of the Swiss study on air pollution and lung diseases in adults. Sozial-Und Praventivmedizin 42 (2):67-84. doi:Doi 10.1007/Bf01318136

McCreanor J, Cullinan P, Nieuwenhuijsen MJ, Stewart-Evans J, Malliarou E, Jarup L, Harrington R, Svartengren M, Han IK, Ohman-Strickland P, Chung KF, Zhang J (2007) Respiratory effects of exposure to diesel traffic in persons with asthma. N Engl J Med 357 (23):2348-2358. doi:10.1056/NEJMoa071535

Menni C, Migaud M, Kastenmuller G, Pallister T, Zierer J, Peters A, Mohney RP, Spector TD, Bagnardi V, Gieger C, Moore SC, Valdes AM (2017) Metabolomic Profiling of Long-Term Weight Change: Role of Oxidative Stress and Urate Levels in Weight Gain. Obesity (Silver Spring) 25 (9):1618-1624. doi:10.1002/oby.21922

Moayyeri A, Hammond CJ, Valdes AM, Spector TD (2013) Cohort Profile: TwinsUK and healthy ageing twin study. Int J Epidemiol 42 (1):76-85. doi:10.1093/ije/dyr207

Polanska K, Hanke W, Jurewicz J, Sobala W, Madsen C, Nafstad P, Magnus P (2011) Polish mother and child cohort study (REPRO_PL)--methodology of follow-up of the children. Int J Occup Med Environ Health 24 (4):391-398. doi:10.2478/s13382-011-0026-y

Polanska K, Hanke W, Krol A, Gromadzinska J, Kuras R, Janasik B, Wasowicz W, Mirabella F, Chiarotti F, Calamandrei G (2017a) Micronutrients during pregnancy and child psychomotor development: Opposite effects of Zinc and Selenium. Environ Res 158:583-589. doi:10.1016/j.envres.2017.06.037

Polanska K, Krol A, Merecz-Kot D, Ligocka D, Mikolajewska K, Mirabella F, Chiarotti F, Calamandrei G, Hanke W (2017b) Environmental Tobacco Smoke Exposure during Pregnancy and Child Neurodevelopment. International Journal of Environmental Research and Public Health 14 (7). doi:10.3390/ijerph14070796

Polanska K, Krol A, Merecz‐Kot D, Jurewicz J, Makowiec‐Dabrowska T, Chiarotti F, Calamandrei G, Hanke WJJoP, Health C (2017c) Maternal stress during pregnancy and neurodevelopmental outcomes of children during the first 2 years of life. 53 (3):263-270

Prpić I, Milardović A, Vlašić-Cicvarić I, Špiric Z, Nišević JR, Vukelić P, Tratnik JS, Mazej D, Horvat MJEr (2017) Prenatal exposure to low-level methylmercury alters the child's fine motor skills at the age of 18 months. 152:369-374

Ricci C, Wood A, Muller D, Gunter MJ, Agudo A, Boeing H, van der Schouw YT, Warnakula S, Saieva C, Spijkerman A, Sluijs I, Tjonneland A, Kyro C, Weiderpass E, Kuhn T, Kaaks R, Sanchez MJ, Panico S, Agnoli C, Palli D, Tumino R, Engstrom G, Melander O, Bonnet F, Boer JMA, Key TJ, Travis RC, Overvad K, Verschuren WMM, Quiros JR, Trichopoulou A, Papatesta EM, Peppa E, Iribas CM, Gavrila D, Forslund AS, Jansson JH, Matullo G, Arriola L, Freisling H, Lassale C, Tzoulaki I, Sharp SJ, Forouhi NG, Langenberg C, Saracci R, Sweeting M, Brennan P, Butterworth AS, Riboli E, Wareham NJ, Danesh J, Ferrari P (2018) Alcohol intake in relation to non-fatal and fatal coronary heart disease and stroke: EPIC-CVD case-cohort study. Bmj-British Medical Journal 361. doi:ARTN k934

10.1136/bmj.k934

Richardson SD, DeMarini DM, Kogevinas M, Fernandez P, Marco E, Lourencetti C, Balleste C, Heederik D, Meliefste K, McKague AB, Marcos R, Font-Ribera L, Grimalt JO, Villanueva CM (2010) What's in the pool? A comprehensive identification of disinfection by-products and assessment of mutagenicity of chlorinated and brominated swimming pool water. Environ Health Perspect 118 (11):1523-1530. doi:10.1289/ehp.1001965

Snoj Tratnik J, Falnoga I, Trdin A, Mazej D, Fajon V, Miklavcic A, Kobal AB, Osredkar J, Sesek Briski A, Krsnik M, Neubauer D, Kodric J, Stropnik S, Gosar D, Lesnik Musek P, Marc J, Jurkovic Mlakar S, Petrovic O, Vlasic-Cicvaric I, Prpic I, Milardovic A, Radic Nisevic J, Vukovic D, Fisic E, Spiric Z, Horvat M (2017) Prenatal mercury exposure, neurodevelopment and apolipoprotein E genetic polymorphism. Environ Res 152:375-385. doi:10.1016/j.envres.2016.08.035

Strumylaite L, Kregzdyte R, Bogusevicius A, Poskiene L, Baranauskiene D, Pranys D (2019) Cadmium Exposure and Risk of Breast Cancer by Histological and Tumor Receptor Subtype in White Caucasian Women: A Hospital-Based Case-Control Study. Int J Mol Sci 20 (12). doi:10.3390/ijms20123029

van Veldhoven K, Kiss A, Keski-Rahkonen P, Robinot N, Scalbert A, Cullinan P, Chung KF, Collins P, Sinharay R, Barratt BM, Nieuwenhuijsen M, Rodoreda AA, Carrasco-Turigas G, Vlaanderen J, Vermeulen R, Portengen L, Kyrtopoulos SA, Ponzi E, Chadeau-Hyam M, Vineis P (2019) Impact of short-term traffic-related air pollution on the metabolome - Results from two metabolome-wide experimental studies. Environ Int 123:124-131. doi:10.1016/j.envint.2018.11.034

Vizcaino E, Grimalt JO, Glomstad B, Fernandez-Somoano A, Tardon A (2014) Gestational weight gain and exposure of newborns to persistent organic pollutants. Environ Health Perspect 122 (8):873-879. doi:10.1289/ehp.1306758

Wright J, Small N, Raynor P, Tuffnell D, Bhopal R, Cameron N, Fairley L, Lawlor DA, Parslow R, Petherick ES, Pickett KE, Waiblinger D, West J, Collaborators BBS (2013) Cohort Profile: The Born in Bradford multi-ethnic family cohort study. International Journal of Epidemiology 42 (4):978-991. doi:10.1093/ije/dys112
